# Optimizing the Synthesis of Deuterated Isotopomers and Isotopologues of Cyclohexene using Molecular Rotational Resonance Spectroscopy

**DOI:** 10.1021/jacs.5c12001

**Published:** 2025-09-12

**Authors:** Justin T. Weatherford-Pratt, Jacob A. Smith, Martin S. Holdren, Haley N. Scolati, Reilly E. Sonstrom, Megan N. Ericson, Sarah E. Brewster, Alvin Q. Meng, Diane A. Dickie, Brooks H. Pate, W. Dean Harman

**Affiliations:** Department of Chemistry, 2358 University of Virginia, Charlottesville, Virginia 22904, United States

## Abstract

Despite advances in reactions such as hydrogen isotope exchange (HIE) and reductive deuteration, achieving controlled and selective deuteration remains challenging. Moreover, the difficulty of developing successful deuteration platforms is compounded by a lack of means to assess the stereoisotopic purity of deuterated products. We previously reported a highly regio- and stereoselective approach for generating semideuterated cyclohexenes via tandem protonation (H^+^/D^+^) and reduction (H^–^/D^–^) sequences of a dihapto-coordinate tungsten-benzene complex. While NMR and HRMS analyses suggested successful deuterium incorporation, molecular rotational resonance (MRR) spectroscopy identified numerous over-, under-, and mis-deuteration impurities. At the time of publication, these impurities were attributed to H/D scrambling that could occur during thermolysis of the tungsten-bound cyclohexene ligand prior to MRR analysis. In this work, we describe the analysis of semideuterated cyclohexenes using MRR spectroscopy with an improved thermolysis apparatus that eliminates deuterium scrambling during analysis. Quantitative analysis of both racemic and enantiopure samples enables the optimization of deuteration conditions by providing multiple mechanistic insights into the formation of impurities.

## Graphical abstract

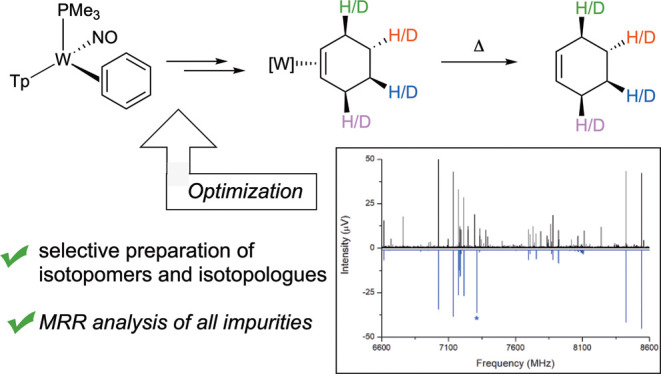

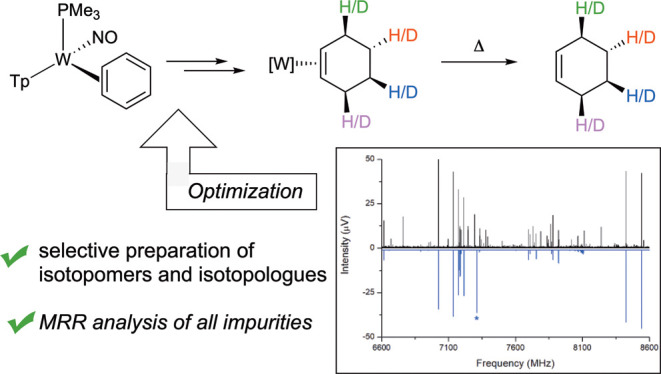

## Introduction

The preparation of deuterated molecules has received considerable attention in recent years due to the advantages gained by replacing a C–H bond with a C–D bond (i.e., the “deuterium switch”). The replacement of hydrogen for deuterium can significantly affect the rate at which the isotopically labeled species react.^1,2^ This observed difference in rates is known as the deuterium kinetic isotope effect (DKIE), and it is the basis for many of the known applications of deuterated molecules—especially in the field of medicinal chemistry, where numerous drugs are metabolized through C–H activation by Cytochrome P450 enzymes.^3,4^ Thus, introducing deuterium at a metabolic site or ″soft spot″ can significantly alter the pharmacokinetics of a drug, providing improved efficacy and valuable mechanistic insight into how metabolites are formed. The benefits of the deuterium switch led to the development of the first FDA-approved deuterated drug, deutetrabenazine, as a treatment of chorea associated with Huntington’s disease.^5,6^ Similarly, the deuterated analogue of sorafenib—donafenib—was recently approved by the National Medical Products Administration of China as a treatment for hepatocellular carcinoma.^7,8^

Given the potential benefits, significant efforts have been made to develop reactions that can efficiently incorporate deuterium into pharmaceuticals.^9^ Current approaches often rely on hydrogen isotope exchange (HIE) via C–H activation or reductive deuteration. A significant challenge with these reactions is to control both the precision of deuteration and the amount of deuterium incorporated, as failure to do so results in mixtures of isotopologues (i.e., isomers differing in isotopic composition) and isotopomers (i.e., isomers differing in isotopic position) that are impossible to separate using chromatography.^10^ Over-deuteration and under-deuteration impurities result in mixtures of isotopologues, whereas the mis-deuteration of a target results in isotopomers. When conducting metabolic studies of a substrate, under-deuteration at the site of metabolism would potentially reduce the effectiveness of the drug. Mis-deuteration or over-deuteration are similarly problematic, as either could induce ″metabolic switching″ to an undesired pathway.^11,12^

Unambiguously identifying deuterated intermediates throughout the manufacture of deuterated active pharmaceutical ingredients (API) is critical to ensuring the API’s final quality.^13^ Yet, optimizing the degree and position of deuteration is a formidable task when the exact isotopic composition of a given reaction mixture is unknown. Standard analytical methods are limited in distinguishing the makeup of isotopic mixtures. For example, Clark et al. recently highlighted some of the limitations of using nuclear magnetic resonance (NMR) spectroscopy in determining the isotopic composition of products formed via a copper-catalyzed transfer hydrodeuteration of arenes.^14^ When analyzing deuterated product mixtures, NMR often fails to differentiate deuterium isotopologues and isotopomers due to signal overlap. Similarly, while high-resolution mass spectrometry (HRMS) can determine which isotopologues are present in a mixture, the technique cannot differentiate between isotopomers. Thus, the development of regio- and stereoselective methods for making deuterated isotopomers as well as methods for accurately determining their structure and percent composition in a mixture are essential to advance this promising area of medicinal chemistry.

New, highly selective approaches to prepare deuterated ″building blocks″ are receiving considerable attention, not only for the preparation of deuterated versions of known compounds, but in the synthesis of de novo pharmaceuticals. While various methods are now available for the selective reductive deuteration of carbonyls, imines, alkenes, and alkynes,^9^ there are few methods for the reductive deuteration of arenes. This is a particularly challenging problem given the potential to create multiple stereocenters in the arene ring. In 2018, Chirik et al. demonstrated a molybdenum-catalyzed reduction of arenes by D_2_ gas (Figure 1, panel A).^15^ However, just as with heterogeneous catalysts, this process yielded complex mixtures of isotopologues and isotopomers owing to H/D scrambling. A highly regio- and stereoselective approach toward making semideuterated cyclohexenes was reported by our group in 2020.^16^ Dihapto-coordination of benzene to the electron-rich {TpW­(NO)­(PMe_3_)} fragment (Tp = trispyrazolylborate) enables protonation (H^+^/D^+^) of the coordinated ligand followed by the addition of a hydride (H^–^/D^–^). A subsequent H^+^/D^+^ and H^–^/D^–^ addition yields various deuterated cyclohexenes, prepared as individual isotopomers (Figure 1, panel B). More recently, a complementary approach was reported by Li et al.^17^ in which hexahapto-coordination of an arene to the electron-deficient {Cr­(CO)_3_} fragment promotes addition of a hydride (H^–^/D^–^) to the coordinated ligand followed by protonation (H^+^/D^+^), thereby affording regio- and stereoselectively deuterated 1,3-cyclohexadienes (Figure 1, panel A).

**Figure 1:**
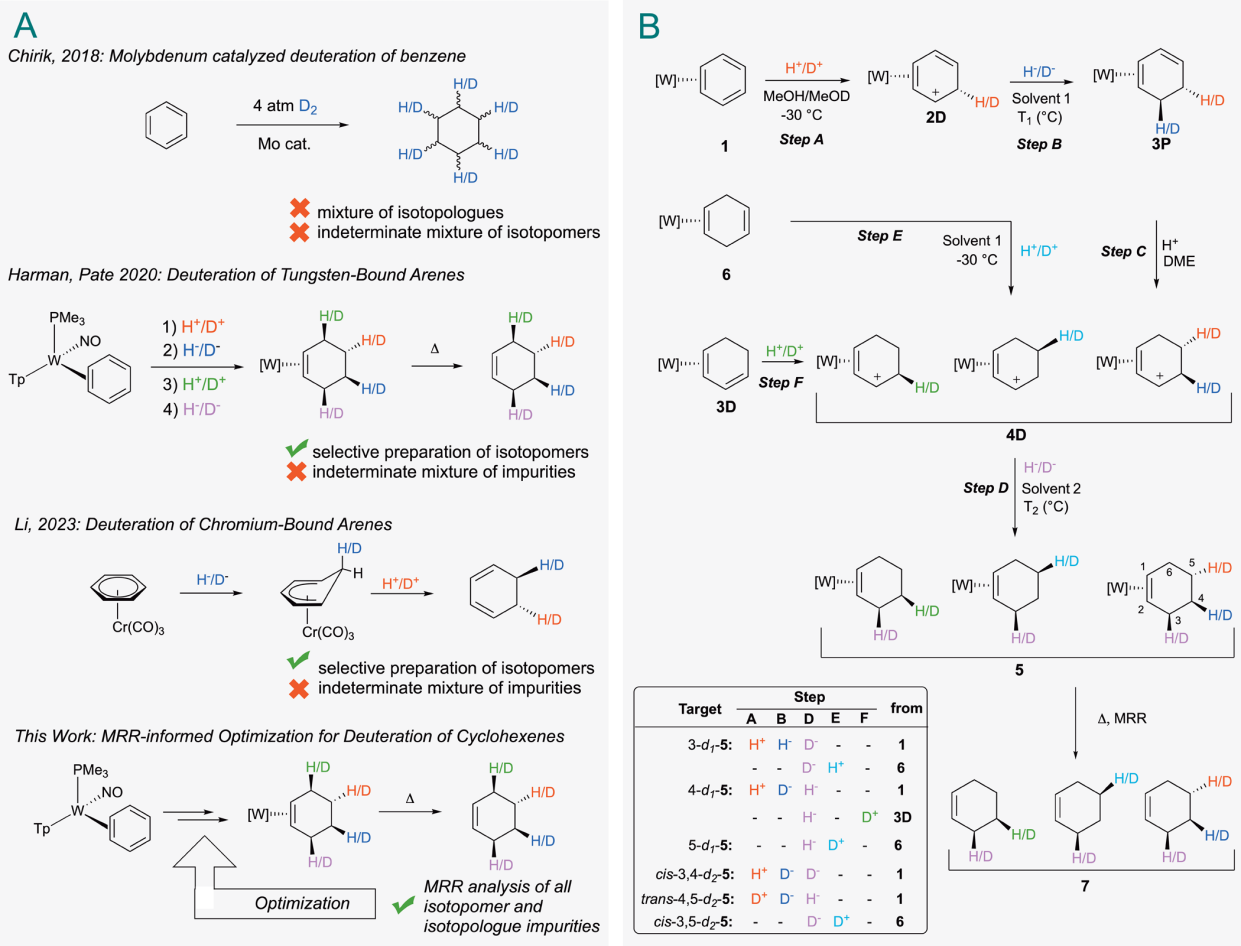
Panel A shows several recent methods for the deuteration of benzene, including the molybdenum catalyzed deuteration of benzene,^15^ deuteration of a tungsten-bound benzene,^16^ deuteration of chromium-bound benzene,^17^ and MRR-assisted optimization of the synthesis of deuterated cyclohexenes. Panel B shows a general synthesis of *d*_1_ and *d*_2_ isotopologues using a chiral tungsten complex. T_1_ and T_2_ are indicated in Figure 3. [W] = {WTp­(NO)­PMe_3_}.

Molecular rotational resonance (MRR) spectroscopy has proven to be a powerful analytical technique for measuring the isotopic purity of deuterated compounds.^14,16–22^ Compared to NMR spectroscopy and HRMS, MRR analysis of deuterated molecules provides a complete description of the isotopic composition—revealing the structural identity and percent composition of a complex mixture of isotopomers. Such an analysis is possible because each distinct isotopomer of a given isotopologue produces a unique rotational spectrum, which can be obtained at such high spectral resolution that spectral overlap is virtually eliminated. The work described in our 2020 report used a combination of quantitative NOE, HRMS, and neutron diffraction data to verify the structure of the isotopomers bound to the chiral tungsten complex {WTp­(NO)­(PMe_3_)} ([W]) and its NO-methylated analog.^16^ MRR analysis of the thermolysis effluent from these complexes was used to unambiguously validate the target structures. However, MRR analysis of the deuterated cyclohexenes revealed significant amounts of under-deuteration and mis-deuteration impurities (i.e., “off-targets”). At the time of publication,^16^ we concluded that these impurities in large part were due to H/D scrambling that was occurring during the thermolysis process, but these impurities obscured our ability to accurately identify the true composition and purity of the cyclohexene isotopomers prior to their liberation.

In this follow-up study, we describe an optimized thermolysis method integrated with MRR analysis that effectively eliminates deuterium scrambling. Hence, impurities still observed may now be attributed solely to the synthetic methodology. Given the capability of MRR spectroscopy to provide highly accurate measurements of isotopic purity, the use of this technique allowed for the optimization of the synthetic process (Figure 1, panel A, bottom scheme). By providing in-depth analysis of all isotopomers generated down to a <1% level, reaction conditions could be tuned to minimize off-target products. Confident that we had eliminated the scrambling issue associated with the measurement, we also endeavored to prepare enantioenriched cyclohexenes rendered chiral by locations of deuterium, and used this information to support or eliminate various mechanistic pathways leading to undesired isotopomers and isotopologues.

## Results

### An Improved Thermolysis Process

The specific goal of this work is to demonstrate how the analysis of isotopic impurities can be used to validate a reaction mechanism and subsequently optimize the synthesis conditions to improve isotopic selectivity. For both goals it is crucial that the deuterated cyclohexene can be removed from the tungsten metal complex without modifying the deuteration pattern created in the reaction sequence. For MRR analysis, cyclohexene is obtained by heating the metal complex sample and using unimolecular thermal dissociation to release the cyclohexene analyte from tungsten. In the initial study,^16^ the sample temperature was limited to 200 °C by the recommended operating conditions of the solenoid valve used to inject sample into the MRR spectrometer. In that work, long sample heating times were required to generate enough cyclohexene sample for analysis. A typical measurement used three successive heating cycles on a single sample loading of 30, 60, and 120 min. The MRR analysis of the liberated cyclohexene showed many isotopic impurities—generally at low levels (1–5%). In many cases, these impurities had no clear connection to the proposed reaction mechanism. For example, it was common to identify isotopic impurities where deuterium had migrated to the CH=CH group. Furthermore, as shown in Figure 2A, the isotopic impurity composition *changed* over the three successive heating cycles of the sample. Given that any liberated deuterated cyclohexene should be stable at 200 °C, these results suggest that at this temperature slow unimolecular isomerization occurs for the cyclohexene that scrambles the isotopologue and isotopomer composition while still bound to the metal.

**Figure 2:**
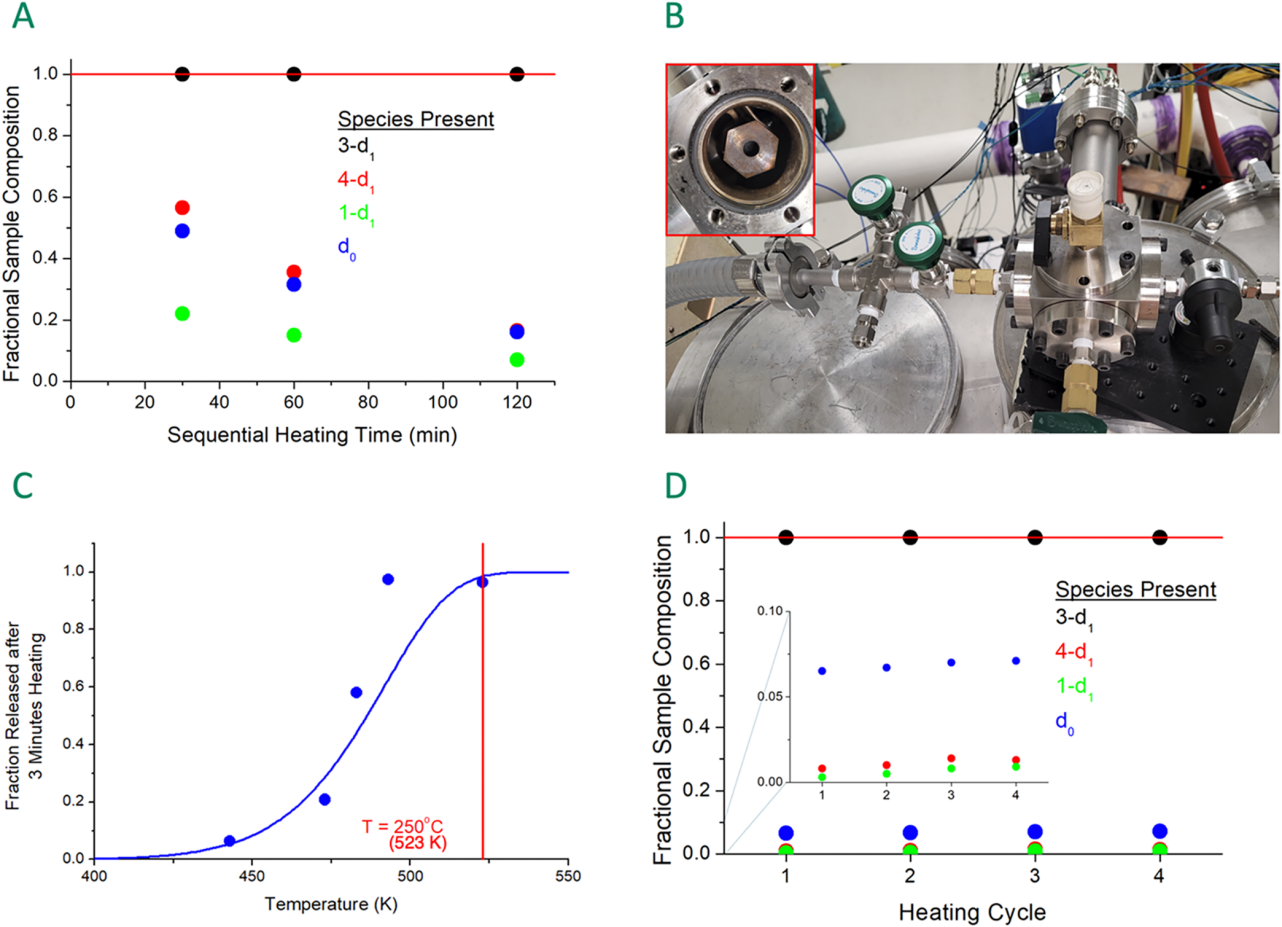
Performance of the new sample system for thermolysis of the tungsten metal complex is illustrated. Panel A shows the time-dependence of the composition of a sample of the 3-deuterocyclohexene complex 3-*d*_1_-**5** after heating in the sample reservoirs of the chirped-pulse spectrometer–the method used in the previous study (ref 16). Panel B shows the external sample system that allows the metal complex to be heated in a stainless-steel crucible (red inset) under a neon atmosphere. The performance of the external sample system is shown in Panel C where the fraction of cyclohexene released from the metal after a 3 min heating period is shown as a function of the final sample temperature. The solid curve is a simple model for the thermal decomposition that will be described in a later publication. Panel D shows the improved performance of the external sample system for the analysis of a 3-*d*_1_-**5** sample. 3-d_1_ = 3-deuterocyclohexene; 4-d_1_ = 4-deuterocyclohexene; 1-d_1_ = 1-deuterocyclohexene; d_0_ refers to proteocyclohexene.

An external sample preparation system for thermolysis of the tungsten complexes, Figure 2B, is used in this work. A detailed description of the sample system and its performance is presented in the SI. By performing the thermolysis outside the spectrometer it is possible to heat the sample to higher temperatures with a faster temperature ramp. The heating conditions for the experiments were determined by measuring the fractional sample decomposition as a function of final sample temperature under conditions where the duration of heating at the final temperature (250 °C) was limited to 3 min in a 1 atm neon gas environment. These results are shown in Figure 2 panel C. The sample loading is typically 30 mg of tungsten metal complex, with a range of 5–50 mg used in the study depending on the amount provided for the analysis. After this time, the sample crucible heater is switched off and the system cools by ambient convection. The sample crucible reaches a temperature of about 140 °C in approximately 10 min. At that temperature, additional neon pressure is added to the sample system to bring the total pressure to 5 atm. This gas mixture is delivered to the spectrometer through a pressure regulator set to 2 atm (15 psig). Each sample preparation in the 0.5 L sample cell provides a mixture that lasts for the measurement of 10,000 rotational free-induction decays averaged in the time domain, measured with a 2–8 GHz chirped-pulse Fourier transform microwave spectrometer at 120 °C. This yields a spectrum with high signal-to-noise ratio and good sensitivity to minor species. Using this external thermolysis system, the sample composition is now observed to be stable over several successive heating cycles as shown in Figure 2 panel D, and the overall impurity level has been reduced.

Measurement throughput is significantly improved using the new thermolysis system and a full measurement cycle can be completed in about 30 min. In contrast, sample analysis was limited to about one sample per day in the previous study due to the time required to access the sample reservoirs inside the spectrometer vacuum chamber for subsequent sample loading. This approach to liberating the cyclohexene in an external sample cell also has the advantage that it is straightforward to add a chiral tag such as propylene oxide to the gas mixture after cooling to 100 °C. In this manner, chiral tag rotational spectroscopy can be performed in order to assign the absolute configuration of cyclohexene enantioisotopomers and measure the enantiomeric excess of the sample.^21^ Chiral analysis of samples prepared using enantioenriched tungsten complexes is shown herein to be a powerful approach to validating the mechanistic source of isotopic impurities in the samples (vide infra).

The MRR analysis results for the liberated 3-deuterocyclohexene (Figure 1 panel B; 3-*d*_1_-**7**) provide a good example of the significant differences between the two sample preparation systems. The sample used for analysis is the 3-deuterocyclohexene complex, 3-*d*_1_-**5** (an explanation of our nomenclature is provided in Figure 1 and further explained below). Upon thermolysis, 3-deuterocyclohexene (3-*d*_1_ *-* **7**) is liberated from the metal and analyzed using MRR. In our initial study,^16^ the MRR analysis of the thermolysis products from 3-*d*_1_ *-* **5** (i.e., isotopomers of type **7**) showed overdeuteration to be <1% relative abundance, but the cyclohexene liberated from the metal contained 12% relative abundance (compared to the major species) of the under-deuterated isotopologue *d*_0_ *-* **7** along with a combined 19% of mis-deuteration impurities in the form of 4-deuterocyclohexene and 1-deuterocyclohexene (4*-d*_1_ *-* **7** and 1-*d*_1_ *-* **7**). These isotopomer impurities have the deuterium migrating one position clockwise and counterclockwise on the cyclohexene ring from the 3-*d*_1_ *-* **7** target, respectively. In an attempt to reduce unexpected impurities in the initial MRR analysis, the nitrosyl ligand of **5** was methylated—a modification that was expected to reduce the ligand-to-metal backbonding, and thus to lower the bond dissociation energy of W-(cyclohexene). In the initial study, this action reduced the level of impurities from 12% to 6% of *d*_0_ and from 19% to 4.5% of mis-deuteration.

Using the improved thermolysis system, which does not require the additional nitrosyl methylation step, the unimolecular dissociation rate was now faster than the rate of hydrogen isotope exchange (HIE), largely responsible for the under-deuterated species *d*_0_-**7**. Higher isotopic purity is observed for the 3-*d*_1_ *-* **7** free cyclohexene liberated from 3-*d*_1_ *-* **5**. The current analysis shows an under-deuteration impurity (*d*_0_ *-* **7**) averaging just 5.7% over three trials and an average of less than 1% of the *d*_1_ isotopomer impurities (4-*d*_1_ *-* **7**, 1-*d*_1_ *-* **7**), with good agreement over three independently prepared samples (3-*d*_1_ Trials 1–3 reported below). It is concluded that the new sample system improves the fidelity of releasing cyclohexene from the tungsten metal complex so that detected isotopic impurities can be attributed more confidently to the reaction chemistry.

### Preparation and Analysis of Racemic Deuterated Cyclohexenes

In our initial report, isotopomers representing ten different isotopologues were prepared (*d*_0_–*d*_4_, *d*_6_–*d*_10_). The synthetic approach to creating these deuteration patterns on cyclohexene uses a common sequence of modular H/D-incorporation steps (Figure 1, panel B). Therefore, quantifying the isotopic impurities and the synthetic pathways that create them for even the simplest deuterated targets can be generalized to any target deuterated cyclohexene. Therefore, we limited this follow-up study to just mono- and dideuteride variations, but with a greater focus on their impurity profiles. The target isotopologues and isotopomers were synthesized by addition of H^+^/D^+^ followed by H^–^/D^–^. Figure 1, panel B depicts the general synthesis of deuterated cyclohexenes (*d*_*n*_ *-* **7**) from benzene as well as from three cyclohexadiene complexes (**3D**, **3P**, **6**). Herein, a deuterated cyclohexene is named according to the relative stereochemistry of deuteriums on the cyclohexene ring (cis or trans), the carbon position deuterated on the cyclohexene ring (1–6), and the number of deuteriums contained on the cyclohexene ring (*d*_*n*_, where *n* = the number of deuteriums). For example, a deuterated cyclohexene with a *cis*-3,4 substitution pattern will be referred to as *cis*-3,4-*d*_2_-cyclohexene or abbreviated as *cis*-3,4-*d*_2_ *-* **7**. Triflic acid (HOTf), *d*_1_-triflic acid (DOTf; 98% D; Sigma-Aldrich), and *d*_2_-diphenylammonium triflate (DPhAT; prepared from DOTf) were used as sources of H^+^ or D^+^. Sodium borohydride (NaBH_4_; 95% CP; Sigma-Aldrich) and sodium borodeuteride (NaBD_4_; 99% D; 95% CP; Cambridge Isotopes) were used as H^–^ and D^–^ sources, respectively. Steps A–D, for which H^+^/D^+^ and H^–^/D^–^ are introduced, are shown for each target (Figure 1, panel B). For example, a 4-*d*_1_ *-* **7** target is accessed through H^+^, D^–^, H^+^, and H^–^ additions at Steps A, B, C and D, respectively. In cases where deuterium incorporation occurs only in the last step (**D**), we found it most convenient to prepare the sample from the 1,4-cyclohexadiene complex **6**, as protonation of this compound directly yields the allyl complex **4D** (Figure 1 panel B).^16^ The reader will note that in Figure 1, panel B, π-allyl complexes are not represented in the conventional manner. Rather, they are depicted as two rapidly interconverting η^2^-allyl complexes with a carbenium carbon either distal (D) or proximal (P) to the PMe_3_ ligand.^16^ Significantly, of the two conformers, the distal is favored by several kcal/mol. A summary of the MRR analyses is given in Figure 3. The relative abundance (relative to the most abundant species) of each species observed and the corresponding reaction conditions are provided. Isotopomers that were identified at less than 1% relative abundance are included in the SI, but not shown in Figure 3.

**Figure 3:**
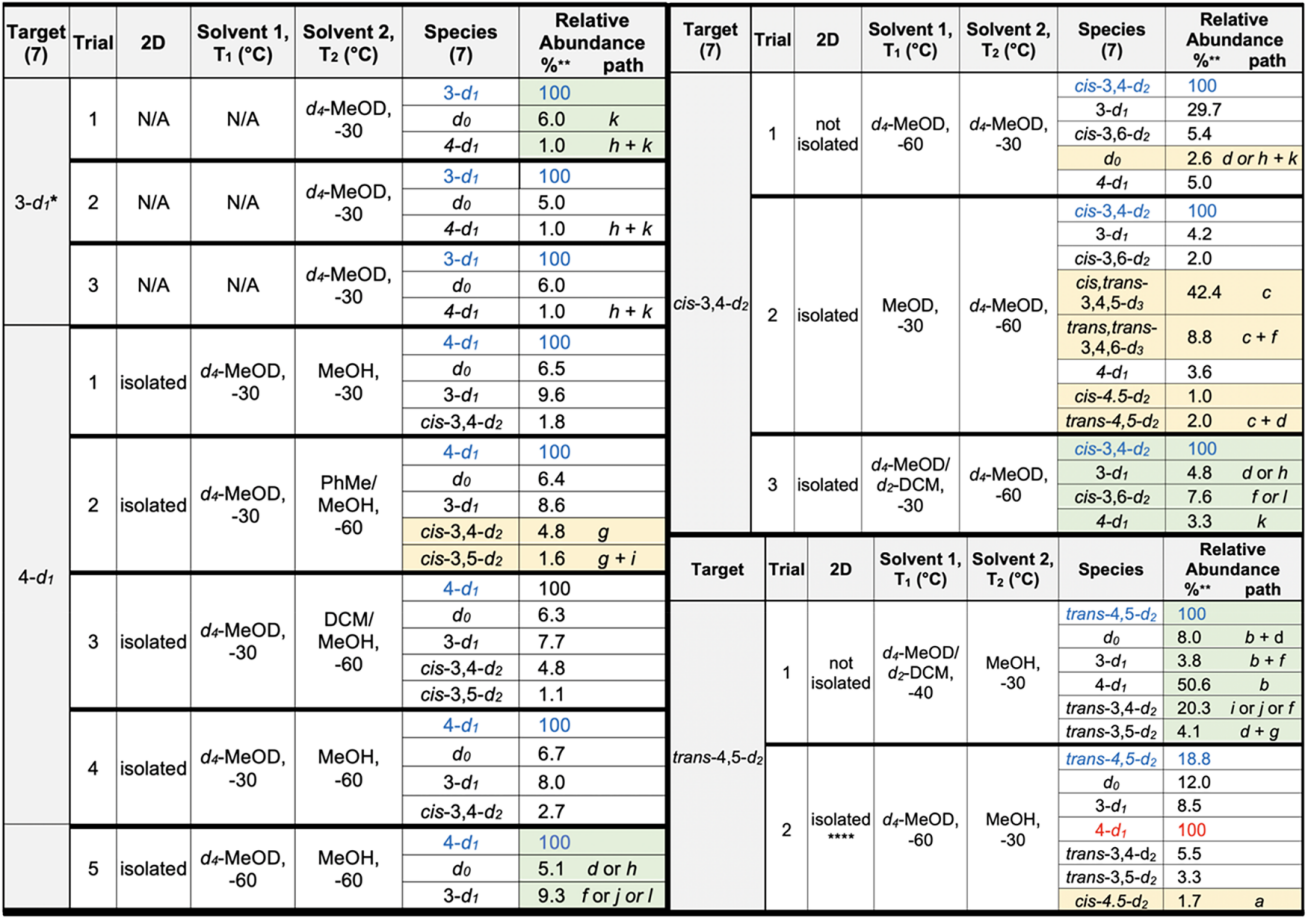
Summary of MRR analysis results for *d*_1_ and *d*_2_ isotopologue syntheses. * 3-*d*_1_-**7** was made from the allyl complex **4D** as prepared from 1,4-cyclohexadiene complex **6**. ** Normalized to relative absolute abundance of target. ****DOTf was used as acid source. Optimized trials are highlighted in green. Impurities eliminated in optimized trial highlighted in yellow. For 4-d_1_, Trial 5: average of three runs, giving a ± 0.8% spread. The designated paths *a-l* refer to Figure 7. Red indicates a dominant product that is not the target.

The 3-deuterocyclohexene complex (3-*d*_1_-**5**) is derived from a deuteride addition to the allyl complex **4D** (Step D, Figure 1, panel B), which can be prepared from the benzene complex **1** or from the cyclohexadiene complexes **3** or **6**. As discussed above, upon releasing the cyclohexene by unimolecular dissociation (3-*d*_1_-**7**), the sample shows high isotopic purity. The main impurity is the nondeuterated cyclohexene (*d*_0_-**7**) that is expected due to reagent impurity of the deuterium source—this impurity is present at an average of 5.7% over the three synthesis trials. All other isotopic impurities are roughly 1% relative abundance or less.

The preparation of 4-deuterocyclohexene complex 4-*d*_1_ *-* **5** requires addition of the deuteride at the benzenium stage (compound **2D**; Step B Figure 1, panel B). Thermolysis of 4-*d*_1_ *-* **5** was not carried out in the initial study,^16^ but ^1^H NMR data from that report suggested high selectivity could be achieved. Thermolysis of 4-*d*_1_ *-* **5** generated 4-*d*_1_ *-* **7** as the major product, however under-deuteration (*d*_0_ *-* **7**) was again present at about 6%. Further, the mis-deuteration product 3-*d*_1_ *-* **7** was observed at the 8–10% level. Also, in contrast to that seen for the 3-*d*_1_ isotopomer, overdeuteration was present, albeit at low levels (*cis-*3,4*-d*_2_ *-* **7**; 1–2%). Lowering the reaction temperature at Step B and Step D from −30 to −60 °C minimized both under-deuteration and overdeuteration (Trial 5; reported data is average of three runs).

The preparation of the complex *cis*-3,4-*d*_2_ *-* **5** introduces deuteriums both at Step B and Step D (Figure 1, panel B). Thermolysis using the new apparatus showed a considerable amount of under-deuterated 3*-d*_1_ *-* **7** (30%) and a small amount of mis-deuterated *cis*-3,6-*d*_2_ *-* **7** (5%) (*Trial 1*). Isolation of the benzenium complex (**2D**) after Step A and a reduced reaction temperature (−30 to −60 °C) at Step D minimized the amount of 3*-d*_1_ *-* **7** (4%) and *cis*-3,6-*d*_2_ *-* **7** (2%) generated (*Trial 2*); however, this resulted in the overdeuterated impurities *cis*-3,4-*trans*-5-*d*_3_ *-* **7** (42%) and *trans*-3,4-*trans*-6-*d*_3_ *-* **7** (9%). Ultimately, it was found that in addition to isolating **2D** and performing Step D at lower temperature, the use of *d*_2_-DCM as a cosolvent in Step B was critical to limiting the formation of 3-*d*_1_ *-* **7** (5%) and *cis*-3,6-*d*_2_ *-* **7** (8%) while also preventing the formation of *cis*-3,4-*trans*-5-*d*_3_ *-* **7** (<1%) and *trans*-3,4-*trans*-5-*d*_3_ *-* **7** (<1%) (Trial 3).

Preparation of *trans*-4,5-*d*_2_ *-* **7** requires introduction of deuteriums at Step A and Step B. Introduction of D^+^ was particularly challenging. The new sample composition analysis of *trans*-4,5-*d*_2_ *-* **7** (Figure 3 *Trial 1*) shows mis-deuteration of the target species: the 24.4% off-target *d*_2_ isotopomers were mostly *trans* configured, with negligible *cis*-isomers (1.1%). However, while overdeuteration was undetected (<1%), high levels of under-deuteration were still an issue, underscoring how important it is to have a large deuterium reservoir in the protonation step.^16^ Unfortunately, isolation of compound **2D** and decreasing the temperature from −30 to −60 °C in Step A (*Trial 2*) increased the amount of the under-deuteration product 4-*d*_1_ *-* **7** to the point that it was the major product. A possible explanation for this observation is that by lowering the temperature, a greater proportion of the proton transfer process occurs through tunneling, whose rate has a less pronounced temperature dependence, compared to the thermal reaction.^23^ The same would not be true for deuteron addition as tunneling is much less prominent. Therefore, even trace (<1%) amounts of a protic impurity (e.g., H_2_O) from commercially available deuterated solvents could significantly affect the isotopic purity of the target cyclohexenes.

As seen in Figure 1, panel B, the π-allyl complex **4D** can be generated by protonation of the benzene-derived proximal 1,3-diene complex **3P**, or from its distal diastereomer **3D**. But remarkably, it also can be formed from the 1,4-cyclohexene complex **6**.^16^ For the latter, DFT calculations support a mechanism involving a nitrosyl-stabilized protonation of the remote alkene (Figure 4, **6H** ^**D**^ or **6H** ^**P**^). We propose that this is followed by a [1,2]-hydride shift of either species to give the π-allyl complex **4D**. The transition states (**6H** ^**D**^ → **4D** and **6H** ^**P**^ → **4D**) were determined to have barriers of about 18 kcal/mol (Figure 4, ). A kinetic barrier of about 32 kcal/mol would seem to eliminate the possibility of the π-allyl complex **4** reverting back to either isomer of **6H**. The analogous process of protonation and hydride-shift *without* participation of the nitrosyl ligand could not be assessed owing to the inability to model the purported isolated secondary carbocation (i.e., **6H** ^**D**^ or **6H** ^**P**^ in Figure 4 without the NO–C bond). While the deuteration of the 1,4-cyclohexadiene **6** was briefly discussed in our initial report,^16^ MRR analysis was not carried out on any of the samples derived in this manner. Hence, the cyclohexene complexes 4-*d*_1_-**7** and *cis*-3,5-*d*_2_-**7** were targeted using the 1,4-cyclohexadiene complex **6** as the precursor. Here, **6** was treated with D^+^, followed by an H^–^ or D^–^ (Figure 4). A summary of the MRR analysis is given in Figure 4. Consistent with previous experiments with D^+^ addition, our attempt to prepare 5-*d*_1_-**5** via the D^+^ addition to the 1,4-cyclohexadiene complex was plagued by under-deuteration: The use of DOTf in *d*_4_-MeOD resulted in a significant level of *d*_0_-**7** (31.9%). This suggests a significant DKIE for the protonation of 1,4-cyclohexadiene complex **6**, much like that observed for the proximal 1,3-diene analogue (**3P**; Figure 1 panel B).^16^ However, MRR analysis also revealed roughly 10% mis-deuteration. In the synthesis of *cis*-3,5-*d*_2_-**5**, using only DOTf or *d*_2_ *-*DPhAT as the deuterium source in CD_3_CN solution also resulted in under-deuteration to the point that 3-*d*_1_ dominated (Trials 1 and 2, Figure 4). However, using *d*_4_-MeOD as the solvent along with *d*_2_ *-*DPhAT significantly decreased the 3-*d*_1_-**7** impurity (15% relative abundance in Trial 3, Figure 4), but also generated ∼ 6% of the *trans-*3,5*-d*_2_ *-* **7** diastereoisotopomer.

**Figure 4:**
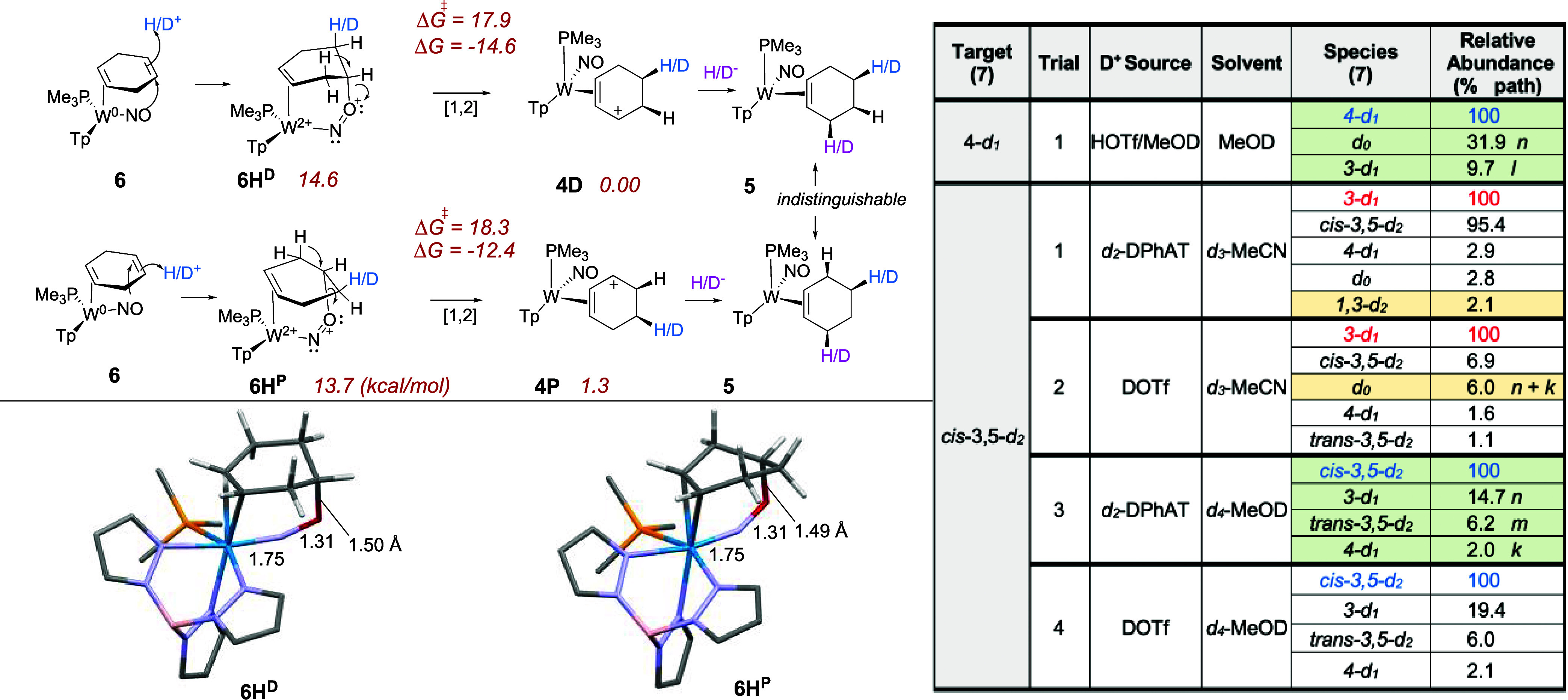
Preparation of 4-*d*_1_-**7** and *cis-*3,5-*d*_2_-**7** via the protonation of 1,4-cyclohexadiene complex **6** and the proposed nitrosyl-stabilized intermediates (**6H** ^**D**^ and **6H** ^**P**^). Optimized trial is highlighted in green. Preparation of 4-d_1_ is from enantioenriched complex **6**. **6H** designiates the protonated form of **6**, where superscript P and D refer to distal and proximal isomers.

### Preparation and Analysis of Enantioenriched Deuterated Cyclohexenes

Enantioenriched products can be obtained using a previously reported enantioenrichment procedure for {WTp­(NO)­(PMe_3_)}.^24^ Previous reports have demonstrated the applicability of this process toward generating chiral organic molecules with little to no observable racemization of the metal center.^24–27^ This procedure involves a trituration step in which longer trituration times improve stereoselectivity but reduce yield. The enantiopurity of the precursor 1,3-dimethoxybenzene (DMB) complex (**8**) was measured by obtaining ^31^P NMR spectra of the corresponding β-pinene diastereomers. Since (*S*)-β-pinene is less than analytically pure (enantiomeric ratio (er) ≈ 49:1), the dr determined via the β-pinene test serves as the lower limit of enantiopurity. Samples of (*S*)-3-*exo-d*_1_-**5** and (*R*)-4-*exo-d*_1_-**5** were derived from the DMB complex **8**, enantioenriched to differing degrees (Figure 5 panel A) via either the 1,3-cyclohexadiene complex **3P** (Trials 2–4) or the 1,4-cyclohexadiene complex **6** (Trial 1).

**Figure 5:**
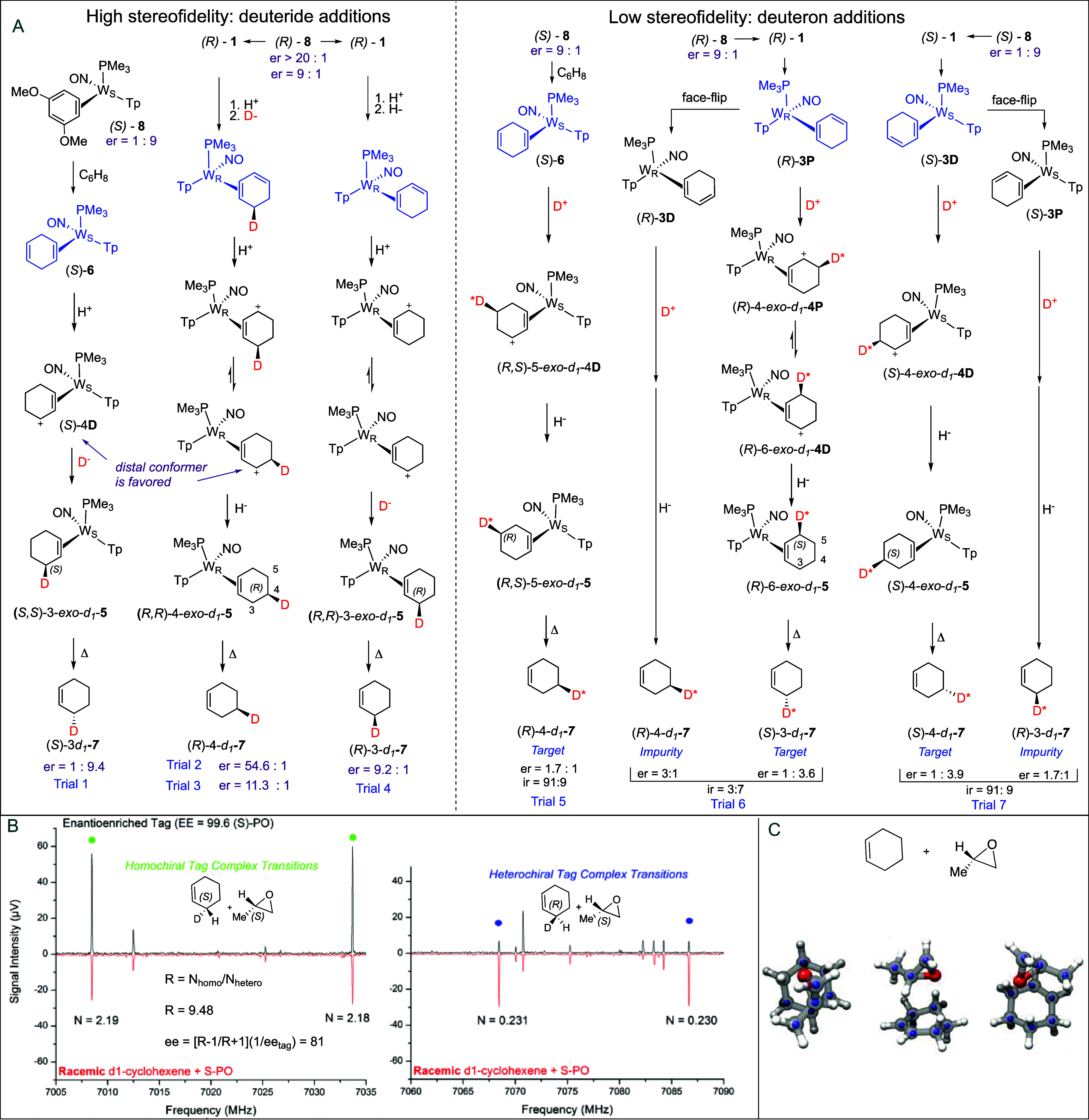
(A) General syntheses of enantioenriched 3-*d*_1_-**7** and 4-*d*_1_-**7**. (B) MRR spectra for both racemic (red) and enantioenriched (black) samples of 3-*d*_1_-**7**. The inset of the top panel shows how the enantiomeric excess is calculated from the intensity ratios of transitions in the homochiral and heterochiral tag complex rotational spectra. Details are presented in the . (C) Three views of the geometry of the chiral tag complex formed between cyclohexene and propylene oxide are shown. The full structure is the equilibrium geometry from quantum chemistry. The smaller blue spheres are the experimental positions of the carbon atoms. D* = poor stereoselectivity.

The enantiopurity of each cyclohexene enantioisotopomer was determined through chiral tag MRR analysis.^28–30^ Our approach uses a traditional enantiomers-to-diastereomers measurement strategy where the analyte enantiomers (which have identical rotational spectra) are differentiated by combining them with an enantiopure chiral molecule (the “tag”). In chiral tag rotational spectroscopy, diastereomeric adducts are created through van der Waals interactions. These adducts are formed in the pulsed jet expansion when a small amount of the tag is added to the inert gas stream used to create the pulsed jet expansion.^31–33^ Significantly, there are no requirements on the position of the tag molecule relative to the stereogenic center(s) of the analyte. Complexation in any position or orientation will generate diastereomeric complexes with distinct, resolved spectra.

Under the Born-Oppenheimer approximation, all deuterated versions of a chiral tag complex with cyclohexene have the same equilibrium geometry and, specifically, share the geometry of the complex formed with the normal isotopic species (i.e., cyclohexene formed with just ^12^C and ^1^H atoms). Therefore, the structure of the chiral tag complex that was used for the chiral analysis can be experimentally validated using the fully proteated species. After screening a few commonly used chiral tag molecules, it was found that the strongest signals were obtained by complexing cyclohexene with (*S*)-propylene oxide (TCI America P3117, ee >97%). The structure of the chiral tag complex used for chiral analysis of cyclohexene is shown in Figure 5 panel C. This geometry is the lowest energy isomer of the weakly bound complex formed between propylene oxide and cyclohexene identified in the computational chemistry study. The spectrum using fully proteated cyclohexene was acquired with sufficient sensitivity that the singly substituted ^13^C isotopomers of the chiral tag complex could be analyzed in natural abundance (SI). This isotopic information was used to determine the experimental carbon atom positions in the molecule using the standard Kraitchman analysis of rotational spectroscopy.^34,35^ The experimental carbon atom positions (smaller blue spheres) are superimposed on the quantum chemistry equilibrium geometry in Figure 5 panel C and validate the accuracy of the theoretical geometry. With an accurate equilibrium geometry, an experimental MRR spectrum can be confidently assigned to a specific deuteration pattern in the chiral tag complex. This spectrum analysis capability is used to assign the absolute configuration of enantioenriched deuterium-labeled cyclohexene species.

Starting from a sample of enantioenriched 1,3-dimethoxybenzene complex WTp­(NO)­(PMe_3_)­(DMB) ((*S*)-**8**; Figure 5; er = 9:1; enantiomeric excess (ee) = 80%), the S enantiomer of 3-deuterocyclohexene was synthesized via the 1,4-cyclohexadiene complex (*S*)-**6**. Upon thermolysis, the cyclohexene was released, combined with the chiral tag (propylene oxide) and evaluated using MRR (Figure 6, Trial 1). A summary of the results obtained from these MRR analyses is shown in Figure 6 (Trials 1–4) where er values for the target compounds and those for observed impurities are given. Additionally, the lower limit of enantiopurity of the starting materials is shown by β-pinene test results.^24^ The method for chiral analysis by MRR spectroscopy is illustrated in Figure 5, panel B where small frequency ranges of the MRR spectra for the homochiral and heterochiral complexes are shown. In this nomenclature, a homochiral tag complex has the same Cahn-Ingold-Prelog designation for the chiral center in both tag and analyte. Measurements using both a racemic (red) and enantioenriched sample (black) of 3-*d*_1_-**7** are presented. In the top spectrum of Figure 5 panel B, the signal intensity for transitions of the homochiral complex are observed to be stronger in the enantioenriched sample compared to the racemic sample measurement. In contrast, the signals of the heterochiral rotational spectrum transitions are weaker for the heterochiral tag spectrum. This indicates that the homochiral tag complexes are present in higher abundance in the enantioenriched sample. Since the tag sample used for the measurements is *(S)*-propylene oxide, the measurement indicates that the absolute configuration of the more abundant enantiomer of the analyte is *(S)*-3-*d*_1_-**7**. After normalization using the transition intensity in the racemic sample measurement, the ee was determined to be 81% ± 2. This corresponds to an er of 9.4:1. Note that the ee of the 3-*d*_1_-**7** sample is nearly the same as the enantioenriched tungsten complex (Figure 6, Trial 1; 3 h trituration time). This suggests that the ee is limited by the initial complex and that the reaction chemistry could have significantly higher stereoselectivity. In a similar manner, when (*R*)-5-*exo*-*d*_1_-**5** was made using highly enantioenriched *(R)-* **1** (prepared from *(R)-* **8**; dr >20:1 via β-pinene test;^24^ 18 h trituration time; see Figure 6, panel B, Trial 2), the target (*R*)-4-*d*_1_-**7** was observed with an er = 54.6:1 (96% ee), in addition to 7% of an (*S*)-3-*d*_1_-**7** impurity (Figure 6, Trial 2). Finally, synthesis of (*R*)-4-*d*_1_-**7** and (*R*)-3-*d*_1_-**7** from the same enantioenriched source (*dr* = 9:1 via β-pinene test; 3 h trituration time) resulted in the desired cyclohexene with er = 11.3:1 and 9.2:1, respectively (Figure 6, Trials 3 and 4).

**Figure 6:**
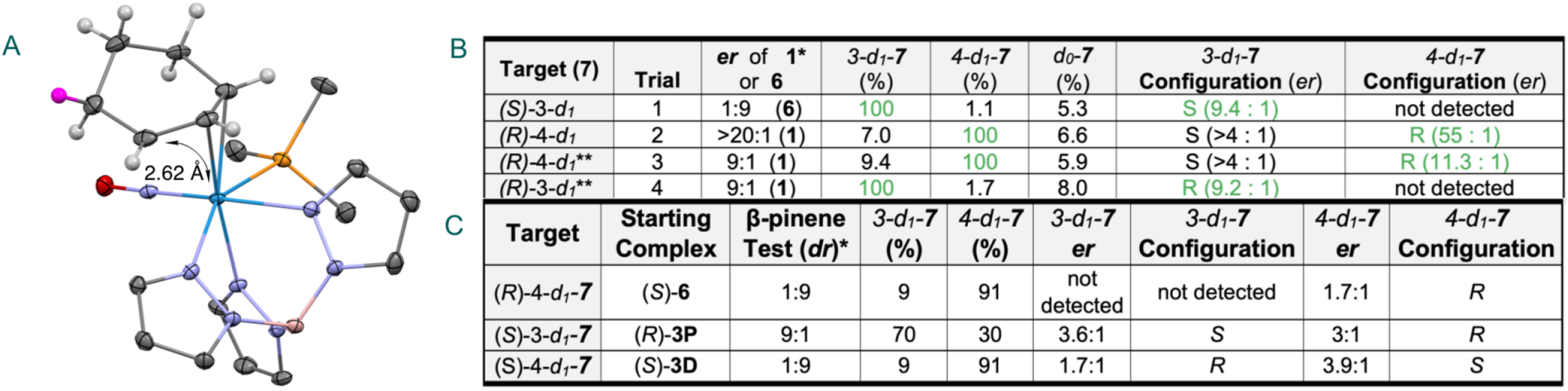
(A) SC-XRD structure of the η^2^ allyl complex (*S*)-4-*exo*-*d*_1_-**4D**; 50% ellipsoids, showing the weak bond to the carbenium carbon (2.62 Å). Absolute configuration determined based on anomalous absorption, with a Flack parameter of −0.015(3). Only one of the two crystallographically independent molecules in the asymmetric unit is shown. Counterions and some hydrogen atoms are omitted for clarity. The deuterium position (shown in pink) could not be determined by diffraction and was estimated through modeling. (B) MRR analysis of enantioenriched samples of 3-*d*_1_-**7** and 4-*d*_1_-**7** as prepared from enantioenriched benzene complex **1** or dimethoxybenzene complex **8**. Trial 1 passed through a 1,4-cyclohexandiene precursor **6**, while Trials 2–4 passed through the 1,3-cyclohexandiene precursor **3P**. (C) 3-*d*_1_-**7** and 4-*d*_1_-**7** as prepared from protonation of a 1,3- and 1,4-cyclohexadiene complexes (**4P**, **4D** and **6**), derived from enantioenriched benzene complex **1**. * ^31^P NMR was used to determine the diastereomer ratio of the corresponding β-pinene complex, as made from DMB complex **8**. ** Made from the same batch of enantioenriched benzene complex **1**.

To extend the enantioenriched deuterium incorporation to protonation of dienes, a series of *d*_1_-isotopomers was synthesized via D^+^ addition to enantioenriched 1,3- and 1,4-cyclohexadiene complexes (*S*)-**6**, (*R*)-**3P**, and (*S*)-**3D**; Figure 5, panel A and their enantiopurity was evaluated using chiral tag MRR spectroscopy. Additionally, whereas up until now the absolute stereochemistry of the metal was determined based on NOE studies of the various isomers of α and β pinene complexes,^24^ we were able to obtain a crystal structure for the allyl complex (*S*)-4-*exo*-*d*_1_-**4D**. This single-crystal X-ray diffraction (SC-XRD) determination (determined based on anomalous absorption, with a Flack parameter of −0.015(3)) confirms the absolute stereochemistry of the metal complex as the *S*-hand (Figure 6, panel A), confirming the conclusions of the original enantioenrichment study.^24^ DOTf in *d*_4_-MeOD was used as the source of D^+^. A summary of the results of the initial determination of the enantiopurity of the starting material, as well as the er values determined through the chiral tag MRR experiments, is given in Figure 6, panel C. The starting material for each synthesis was enantioenriched to the same degree (*dr* = 9:1 via β-pinene test;^24^ 3 h trituration time). In stark contrast to the high enantioenrichment obtained when deuterium was introduced as a deuteride (D^–^), chiral tag MRR analysis revealed that deuteration of (*S)*-**6** resulted in the cyclohexene (*R*)-4-*d*_1_ *-* **7**, formed in only a modest enantiomeric excess er = 1.7:1. Similarly, when 1,3-cyclohexadiene complexes (*R*)-**3P** and (*S*)-**3D** were deuterated, the cyclohexenes *(S)-*3-*d*_1_-**7** and *(S)-*4-*d*_1_-**7** were generated with only partial enantioenrichment (er = 3.6:1 and 3.9:1, respectively). Additionally, a large amount of (*R*)-4-*d*_1_-**7** (30%) was observed in the synthesis of (*S*)-3-*d*_1_-**7**. Together these observations indicate that while D^+^ addition to the η^2^-benzene complex occurs *syn* to the metal, and with high selectivity (∼9:1),^16^ D^+^ addition to the η^2^-diene complexes **3D** or **3P** preferentially occurs *anti* to the metal, and is only modestly stereoselective. In addition, the acidic conditions appear to partially induce a “face-flip” of the diene (Figure 5), which results in constitutional isotopomers (3-deutero- and 4-deuterocyclohexene isomers). Curiously, a similar reaction sequence to form *(S)-*3-*d*_1_-**7** from the 1,4-cyclohexadiene complex **6** involving H^+^ did not compromise the enantioenrichment nor the constitutional purity (Figure 6, panel B Trial 1). This is consistent with the notion that there is an unusually large DKIE in play for the protonation of diene complexes.^16^

## Discussion

Given the rapid interconversion of the ring conformations at ambient temperatures, the ^1^H NMR spectrum of cyclohexene has three signal-averaged peaks in a 2:2:1 ratio at 25 °C. Consequently, the four isotopomers cis-3,4-, cis-3,5-, trans-3,4- and trans-3,5-dideuterocyclohexene, for example, have indistinguishable ^1^H NMR spectra making an analysis of their mixture virtually impossible. In our initial report,^16^ HRMS was used to estimate the isotopic purity of cyclohexene isotopologues and ^1^H NMR data allowed us to analyze various cyclohexene isotopomers when complexed to [WTp­(NOMe)­(PMe_3_)]^+^. Such complexation allowed almost complete resolution between the 10 distinct cyclohexene signals, yet overlap issues of signals corresponding to protons away from the asymmetric metal center were still problematic, and detailed analysis of mixtures, especially of impurities at low relative abundance, were beyond reach. MRR analysis coupled with an optimized thermolysis process revealed that what was hiding beneath an otherwise “clean” NMR spectrum of a targeted species was in fact a range of isotopomer impurities, typically well under 10% relative abundance compared to the target. Collectively, the pattern of under-, over-, and mis-deuterations revealed unexpected reactivity pathways that could be partially mitigated or modulated to optimize the purity of a given target. Based on the nature and quantity of the isotopomer and isotopologue impurities for each target, and how their distribution is affected by changes in reaction conditions, a universal set of off-target reaction pathways was identified (**a**–**n** in Figure 7) that are responsible for most impurities generated through Steps A–E in Figure 1, panel B. A summary of these off-target reaction pathways follows.

**Figure 7:**
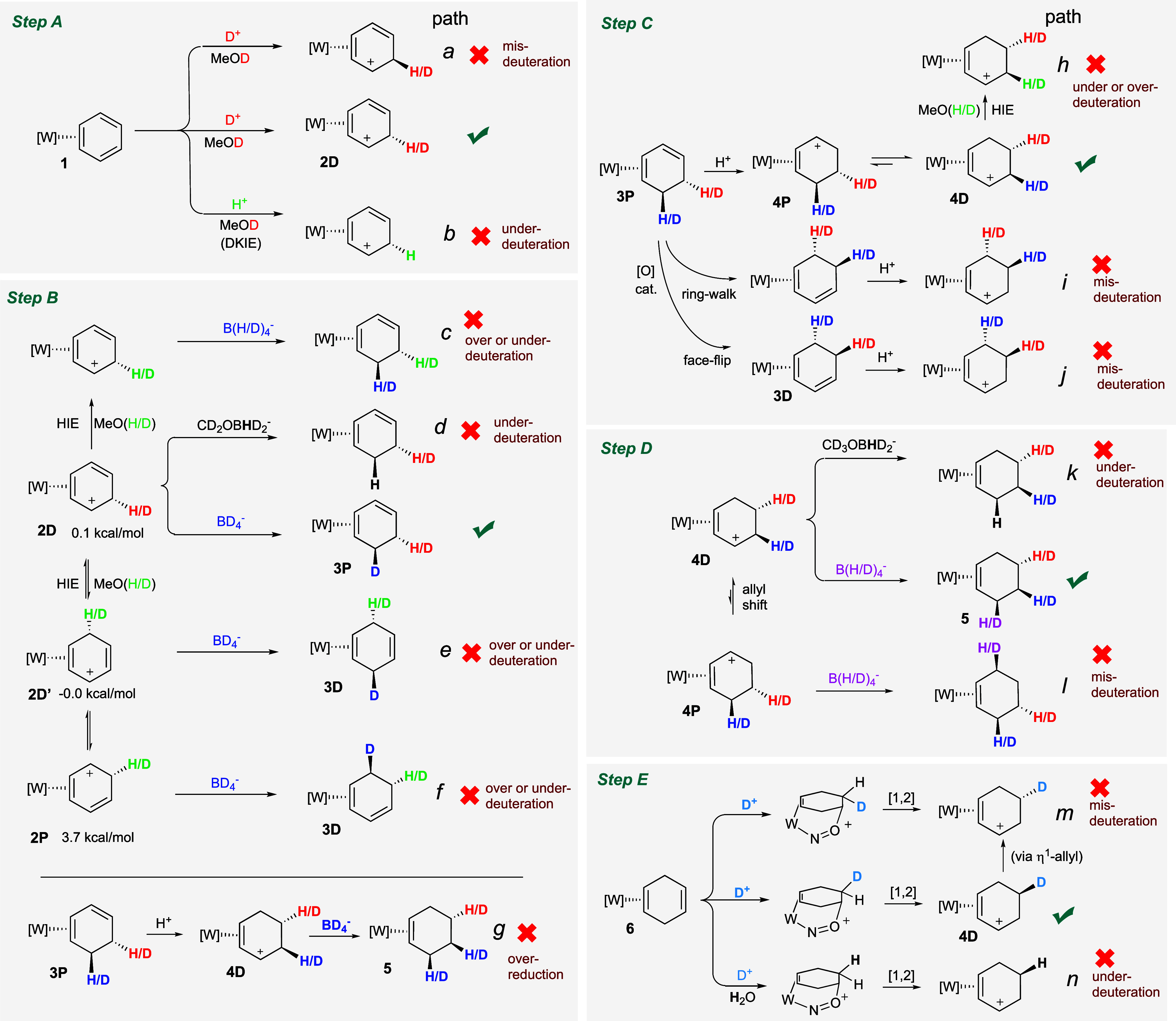
Off-target reaction pathways (a–n) for Steps A–E.

### Under-Deuteration (Paths *b, d, h, k, n*)

A significant amount of the impurities in Figure 3 come from under-deuteration, which manifests in two ways: hydride addition instead of an intended deuteride addition, and H^+^ addition rather than an intended D^+^ addition (Figure 7). Regarding the former, as BD_4_^–^ starts to react in CD_3_OD, it forms the intermediate CD_3_OBD_3_^–^,^36,37^ which is more reactive than the initial borodeuteride salt. It also, we suspect, is more prone to H/D exchange with adventitious water, such as has been documented for BH_3_NMe_3_.^38^ The DKIE of borohydrides is likely to have a significant amplifying effect.^39^ Thus, in the current experiments we see 3–7% under-deuteration in reactions involving BD_4_^–^ (pathways **d**, and **k**) in Steps B and D. This results in the 3-*d*_1_-**7** and 4-*d*_1_-**7** impurities, respectively, for the preparation of *cis-*3,4-*d*_2_-**7**, and the *d*_0_-**7** impurity in the preparations of 3-*d*_1_-**7** and 4-*d*_1_-**7**. For reactions involving D^+^, (Steps A, E; paths **b**, **n**) impurities can be significantly higher, especially when it comes to protonation of a tungsten η^2^-diene complex (vide supra). We have observed a nonclassical DKIE as high as 40 in the case of diene complex **4P**,^16^ and thus rigorously dry reaction conditions are required to avoid under-deuteration impurities from being the dominant species (e.g., Trial 2 of *4,5-trans-d*_2_-**7**). For these reasons, we did not revisit D^+^ addition in Step C.^16^ Finally, under-deuteration can presumably occur from acidic arenium and allyl species (**2** and **4**; path **c**, **f** and **h**; green hydrogens) undergoing D/H exchange with a protic solvent. For example, path **h** would offer an alternative to path **d** to explain the appearance of 3-*d*_1_-**7** in the preparation of *cis-*3,4-*d*_2_-**7**.

### Over-Deuteration (Paths *c, g, h*)

Overdeuteration was seldomly observed for the reaction sequence in Figure 1, panel B. When it did occur, it was primarily by HIE (path **c** or **h** in Figure 7) of acidic species in deuterated solvents. One example where this reaction was significant was Trial 2 of *cis-*3,4-*d*_2_-**7**. Overdeuteration can also can occur by an over-reduction Path **g** in which the product cyclohexadiene complex of the first reduction partially protonates and is reduced a second time. Such a reaction seems to occur only at very low levels (typically under 2%). Finally, we note that in cases where there is over- or under-deuteration, the selectivity appears to be remarkably high. For example, for *cis-*3,4-*d*_2_-**7**, the only overdeuteration impurities at or above a 1% level are the two *d*_3_ isotopomers *cis-trans*-3,4,5-*d*_3_-**7** and *trans-trans-*3,4,6-*d*_3_-**7**, and these showed up only in Trial 2. There was no hint of the other 28 isotopomers (racemic) possible for *d*_3_-cyclohexene. For the synthesis of *trans-*4,5*-d*_2_ *-* **7**, there were no *d*_3_-**7** isotopomers present at all. Regarding the under-deuteration of these compounds, 4-*d*_1_-**7** and 3-*d*_1_-**7** were present, but there was no infiltration of the alkene by deuterium.

### Mis-Deuteration (Paths *a, e, f, j, i, m, l*)

Mis-deuteration was present in all samples to varying degrees, but similar to overdeuteration, the impurities tended to be very specific, lending themselves to mechanistic analysis (Figure 7). The most common form of mis-deuteration was at the deuteride addition Step B and Step D. These mis-deuterations appear to be stereoselective, but differ in location compared to the intended target of the cationic arenium or allyl ligand. These include paths **e** and **f** for Step B (Figure 7), and path **l** for Step D. Alternatively, protonation at the diene and arene stages can result in a mis-deuteration in a subsequent hydride addition. These reaction pathways include the face-flip or ring-walk of the diene triggered by an adventitious oxidant (Step C, path **i** or **j**),^40^ or paths **e** and **f** (Step B), which involve a mis-protonation that in turn leads to mis-deuteration. Finally, a mis-protonation of 1,4-cyclohexadiene can lead to mis-deuteration in Step E (vide infra).

While several of these reaction pathways lead to similar products, they can be differentiated when absolute stereochemistry is considered. For example, the most common mis-deuteration for 4*-d*_1_-**7** is 3*-d*_1_-**7**, but this could occur by several different mechanisms (please see Figure 5). In Figure 6, results are shown for the enantiopurity and absolute stereochemistry of the deuterated cyclohexene for both the target 4*-d*_1_-**7** and the impurity, 3*-d*_1_-**7**. In all cases the absolute stereochemistry of the dominant species matched expectation according to the reaction scheme in Figure 1, panel B. Particularly revealing, however, was the absolute configuration of the dominant impurity. When *(R)-*4-*d*_1_ was prepared (D^–^ in Step B) from *(R)-* **1**, the impurity 3-*d*_1_-**7** was determined to be the S configuration (er >4:1). This observation rules out the mis-deuteration occurring at the allyl stage (Step D, path **l**, where a H^–^ adds to allyl **4P** rather than **4D**) as it would have resulted in the R configuration of the 3-*d*_1_-cyclohexene (*(R)-*3-*d*_1_-**7**; Figure 5). One also could envision a reaction pathway where the misdirect event is the diene complex **3P** undergoing a “face-flip” isomerization to form **3D** (Step C, path **j**). Subsequent protonation and hydride addition would again form *(R)*-3-*d*_1_-**7** (Figure 5). This leaves path **f** in which the D^–^ adds to the minor isomer of the arenium complex **2P** (Figure 7) in Step B. Similar behavior was observed for the addition of other nucleophiles to the benzenium complex.^25^ In this event, the new stereocenter is formed in an S configuration. Upon subsequent protonation and H^–^ addition, the product would be the observed *(S)*-3-*d*_1_-**7**. The observation that the intended product *(R)-4-d*_1_-**7** was prepared with an enantiomer ratio of 55:1 also rules out a “ring-walk” isomerization of the diene complex (Step C, pathway **i**) from significantly competing with the desired reaction pathway, as this would have produced the opposite enantiomer.

Another example of how absolute configurations of isotopomers can inform mechanistic considerations comes from the preparation of (*S)-*3*-d*_1_ *-* **7** starting from the proximal diene complex (*R)-* **3P** (Figure 5). While the regioselectivity is not high (isomer ratio: ir = 7:3), the desired *(S)-*3*-d*_1_ -**7** cyclohexene stereoisomer is the major product. Significantly, the byproduct is (*R)-4-d*_1_-**7**, which indicates that a “face-flip” of the diene (Step C, path **j**; Figure 7) must have occurred from (*R)-* **3P** to (*R)-* **3D.** We have previously observed this face-flip event occurring in the presence of adventitious oxidants.^40^ Interestingly, the constitutional isomer ratio of 4-*d*_1_-**7**: 3-*d*_1_-**7** is much better for the distal diene complex **3D**, where ir = 91:9 (Figure 6, panel C). And when *(S)-*4-*d*_1_-**7** was targeted starting from *(S)-* **3D**, the *R* enantiomer of 3-*d*_1_-**7** was the major stereochemical impurity. As with the opposite hand, the face-flip of diene *(S)*-**3D** to *(S)-* **3P** appears to be the mechanism for the formation of *(R)-*3-*d*_1_-**7** (path **j**). Finally, we note that in contrast to the hydride additions, stereoselectivity of D^+^ addition to diene complexes is relatively poor, and this explains the rather low enantiomeric excess for all of the cyclohexenes in Figure 5 derived from dienes, where the enantiomer ratio ranges from 1.7:1 to 3.9:1. In particular, a protonation *anti* to the metal of the 1,4-cyclohexadiene **6** at either alkene carbon would result in the target *(R)*-4-*d*_1_-**7**, thus, the observation of a low ee for this compound suggests a poor syn:anti protonation ratio. However, for experiments concerning the synthesis of *cis-*3,5-*d*_2_-**7**, only a 6% trans impurity was observed (Figure 4, Trials 3–4), so it is possible that the acid may be scrambling the metal stereocenter in this case. What is noteworthy is that the impurity 3-*d*_1_-**7** is detected in roughly the same ratio as it was when the 4-*d*_1_-**7** target was prepared from benzene. Both reaction pathways pass through a common intermediate of an allyl complex with a deuterium at C5, and it is possible that another misdirect pathway exists. Short of this, the isomerization would have to occur via deprotonation to form a 1,3-diene intermediate followed by a ring-walk (pathway **i**) followed by reprotonation which would place the deuterium in the 3 position.

In addition to a large amount of under-deuteration, MRR analysis of the *trans*-4,5-*d*_2_-**7** target show mis-deuteration (*trans*-3,4-*d*_***2***_-**7**; 20%). While in principle this could be attributed to either a misdirect at the allyl stage, (path **l**) or a result of either a ring-walk (path **i**) or face-flip (path **j**), the information from analysis of enantioenriched samples of benzene complex **1** indicate that pathway **f** is most consistent with the observations, where the deuteride adds to the proximal isomer of the arenium, **2P**. While pathways **i**, **j**, and **l** would provide the observed *trans*-3,4-d_2_-**7** impurity, we know from the above experiments targeting *(R)-*4-*d*_1_-**7** that pathway **f** must be a major contributor to this impurity (Figure 6, panel B).

As a general comment, the cis/trans selectivity in the deuteration of benzene is exceptional, where the ratio for the target *cis-*3,4-*d*_2_-**7** to all *trans* impurities is over 100:1 in the optimized runs (Figure 3; green). Likewise, the ratio of the target *trans-*4,5-*d*_2_-**7** to all *cis* impurities is over 100:1. The one case where we see a lower ratio in optimized conditions comes in the targeted synthesis of *cis-*3,5-*d*_2_-**7** from the 1,4-cyclohexadiene complex **6**, where even under optimized conditions we detect 6% of the *trans* stereoisomer. In this case, there appears to be somewhat less stereoselectivity in the protonation of the isolated alkene bond (path **m**; Figure 7), consistent with our earlier conjecture. A similar outcome was observed in our initial study where ^1^H NMR data indicated that deuteration *syn* to the metal of the diene complex **3P** could be as high as 20% (cf. 80% anti deuteration).^16^ While this *trans-*3,5-*d*_2_-**7** stereoisotopomer impurity could also be formed from a face-flip at the allyl stage (**4**) via an η^1^ allyl intermediate (path m, Figure 7), such a mechanism would have compromised the stereofidelity of *cis-*3,4-*d*_2_-**7** and *trans*-4,5-*d*_2_-**7** (no stereoisotopomer impurities were observed).

As illustrated above, MRR analysis was an invaluable tool in optimizing synthetic procedures. In the first trial targeting *cis*-3,4-*d*_2_-**7**, the benzenium complex, **2D**, was formed *in situ* and immediately treated with BD_4_^–^, resulting in a significant amount of the under-deuteration product 3-*d*_1_-**7**. We suspected that this was the result of protic sources, such as MeOH and HOTf, decreasing the isotopic purity of NaBD_4_. Thus, a competitive H^–^ addition occurs to **2D** instead of the desired D^–^ addition (path **d**). To test this hypothesis, **2D** was isolated after its preparation in MeOH prior to its treatment with D^–^. Gratifyingly, the 3-*d*_1_-**7** impurity was drastically reduced from 30% to 4%. However, new overdeuteration impurities resulted upon thermolysis, including *cis,trans*-3,4,5*-d*_3_-**7** and *trans,trans*-3,4,6*-d*_3_-**7**. Due to the acidic nature of arenium **2D** (p*K*_a_ 1–2),^25,41^ we postulated that in *d*_4_-MeOD, H/D exchange could occur at C6 (path **c**). Subsequent D^–^ additions would result in the observed *cis,trans*-3,4,5*-d*_3_-**7**, and a similar pathway from the proximal isomer **2P’** would form *trans,trans*-3,4,6*-d*_3_-**7** (path **c + f**). Thus, we sought to minimize exposure of **2D** to H/D exchangeable solvents by first dissolving **2D** in *d*_2_-DCM before adding it to a chilled mixture of NaBD_4_ in *d*_4_-MeOD. The optimized procedure for the synthesis of *cis*-3,4-*d*_2_-**7** effectively minimized under-deuteration and eliminated overdeuteration (Trial 3; Figure 3). To highlight the analytical complexities if one were to use ^1^H NMR spectroscopy alone, consider Trial 1 of the preparation of the tungsten complex *cis*-3,4-*d*_2_-**5**. A 30% impurity of 3-*d*_1_-**7** would result in a 30% signal of the signal at 2.93 ppm. However, this same signal intensity could be a result of *d*_0_-**7**, 4-*d*_1_-**7**, 4,5-*d*_2_-**7** or other isotopic impurities. The 4-*d*_1_ *-* **7** impurity of 5% would be even more difficult to detect as H4 and H5 signals overlap even when the cyclohexene is complexed to the asymmetric tungsten fragment.^16^

A summary of mechanistic insights concerning the chemistry outlined in Figure 1, panel B follows:

1. Deuteride (D^–^) addition is highly stereoselective (>99%) for *anti*-addition to the metal, both for the η^2^-arenium complex **2**, and the η^2^-allyl complexes **4P** and **4D**.
2. Deuteron addition (D^+^) to the benzene complex is highly stereoselective (>99%), favoring *syn*-addition to the metal.
3. Deuteron addition (D^+^) to any of the η^2^-diene complexes (**3P**, **3D**, and **6**) is stereoselective for *anti*-addition (>99% for **3P** and **3D**, and >90% for **6**).
4. Mis-deuteration occurs primarily as a result of poor regioselectivity of H^–^/D^–^ addition to the η^2^-arenium species **2**.
5. Mis-deuteration resulting from addition to the proximal form of the η^2^-allyl species (**4P**) is minimal.
6. For any D^+^ addition, even low relative concentrations of H^+^ successfully compete, resulting in significant under-deuteration. We attribute this to an unusually high DKIE (∼40).
7. BD_4_^–^ can react with trace amounts of H_2_O to form active hydride reducing agents, resulting in under-deuteration.
8. Acidic η^2^-arenium and η^2^-allyl species can undergo exchange with protic solvents resulting in under-deuteration.
9. Incorporation of deuterium into the alkene portion of the cyclohexene is virtually nonexistent (<1%) using the methods described herein.

MRR spectroscopy’s ability to accurately determine the isotopic composition of deuterated cyclohexenes makes it an invaluable tool for optimizing the synthesis of such compounds, even in cases where the analytes have relatively low vapor pressure (e.g., methylphenidate).^42,43^ Rotational analysis revealed impurities virtually impossible to identify or differentiate by NMR spectroscopy. While it was initially thought that mis-deuteration impurities arose through a single pathway, chiral-tagging MRR experiments revealed an alternative route to mis-deuteration at the arenium stage that would have been difficult to support without such definitive data. MRR studies also helped identify the conditions necessary to maximize the selective incorporation of D^+^ into dienes coordinated to the {TpW­(NO)­(PMe_3_)} fragment by exploring isotopologue and isotopomer distributions that result when using different acids. The stereoselectivity of D^+^ incorporation was also evaluated by measuring the enantiomeric excess of chiral isotopomers made via deuterating enantioenriched dienes. Finally, chiral tagging experiments provided validation of absolute configuration and quantification of enantiopurity. More generally, this work demonstrates how MRR analysis can aid in mechanistic analysis and in minimizing or eliminating impurities commonly encountered during the precision synthesis of deuterated compounds.

## Supplementary Material





## Data Availability

Correspondence and requests for materials should be addressed to wdh5z@virginia.edu. All data is available in the main text or the . CCDC Deposition Number 2350579 contains the supplementary crystallographic data for this paper. These data can be obtained free of charge from The Cambridge Crystallographic Data Centre via www.ccdc.cam.ac.uk/structures. The rotational spectra, full spectroscopy fit results, and chiral tag rotational spectroscopy analysis data are available through Zenodo 10.5281/zenodo.15548822.

## Notes

DFT Optimized structures (XYZ)
Full synthetic procedures, NMR spectra, compound characterizations, DFT and MRR analysis (PDF)
