# Supplementary material for: Optimizing the Synthesis of Deuterated Isotopomers and Isotopologues of Cyclohexene using Molecular Rotational Resonance Spectroscopy
Source: J Am Chem Soc. 2025 Sep 12;147(38):35055–68. doi: 10.1021/jacs.5c12001 (PMC12464997; doi:10.1021/jacs.5c12001)
Supplement: Supplementary file 2 [file ja5c12001_si_002.pdf]

Supporting Information

Optimizing the Synthesis of Deuterated Isotopomers and Isotopologues of Cyclohexene using Molecular Rotational Resonance Spectroscopy

Justin T. Weatherford-Pratt,[1] Jacob A. Smith, [1] Marty Holdren, [1] Haley N. Scolati, [1] Reilly E. Sonstrom, [1] Megan N. Ericson, [1] Sarah E. Brewster, [1] Alvin Q. Meng, [1] Diane A. Dickie, [1] Brooks H. Pate,*, [1] and W. Dean Harman*,[1]

[1] *Department of Chemistry, University of Virginia, Charlottesville, Virginia, USA 22904*

Corresponding author e-mails: Brooks H. Pate bp2k@virginia.edu W. Dean Harman: wdh5z@virginia.edu

Table of Contents

Synthetic Methods S2

General Methods S2

General Procedure 1 .... S2

General Procedure 2 .... S2

Representative Optimized Syntheses of Deuterated Cyclohexenes S2

Synthesis of 2D S2

Synthesis of 6-d$_1$-2D S2

Synthesis of 3P S3

Synthesis of 3D S3

Synthesis of 5-d$_1$-3P S3

Synthesis of 4D S4

Synthesis of 6-d$_1$-4D S4

Synthesis of 5-d$_1$-4D S4

Synthesis of 4,5-trans-d$_2$-4D S4

Synthesis of 3-d$_1$-5 .... S5

Synthesis of 4-d$_1$-5 .... S5

Synthesis of cis-3,4-d$_2$-5 .... S5

Synthesis of trans-4,5-d$_2$-5 .... S5

Synthesis of cis-3,5-d$_2$-5 .... S5

NMR Spectra S6

Supporting Information on Molecular Rotational Spectroscopy Analysis S7

Crystallographic Data S40

DFT calculations S42

Synthetic Methods

General Methods: NMR spectra were obtained on a 600 or 800 MHz spectrometer. Chemical shifts are referenced to tetramethylsilane (TMS) utilizing residual ^{1}H or ^{13}C signals of the deuterated solvents as internal standards. All synthetic reactions were performed in a glovebox under a dry nitrogen atmosphere unless otherwise noted. All solvents were sparged with nitrogen prior to use. Reagents were purchased from commercial vendors and used as received without purification. The transition-metal fragment WTp(NO)(PMe_3), where Tp = trispyrazolylborate, is abbreviated as [W]. Enantioenriched samples were prepared under identical conditions to their racemic analogues, however, ***(R)*-1**, ***(S)*-1**, ***(R)*-6** or ***(S)*-6** was used as the starting material. [1-2] These four compounds were all derived from enantioenriched 1,3-dimethoxybenzene complex.[3]

General Procedure 1: To 30-mL test tube was added **1** and DCM or *d_2*-DCM. In a separate 15-mL test tube was added MeOH or MeOD and HOTf or DOTf. Both test tubes were chilled to -30 °C for 15 minutes before the solution of acid was added to the solution of **1**. The solution was allowed to stir at -30 °C for 30 minutes before adding to cold (-30 °C) stirring Et_2O (300 mL). The resulting dull-yellow precipitate was collected on a 60-mL M frit and washed with 50 mL (2 x 25 mL) of cold Et_2O.

General Procedure 2: To 30-mL test tube was added **4P** and MeOH or MeOD. The resulting solution was chilled to -60 °C for 5 minutes before slowly adding $NaBH_4$ or $NaBD_4$. After one hour, the reaction mixture was then diluted with DCM (5 mL) and washed with DI H_2O (3 x 5 mL). The organic layer was dried over anhydrous $MgSO_4$ and concentrated in vacuo. The resulting film was dissolved in minimal DCM and added to stirring pentanes. The solution was evaporated down to approximately ¼ the initial volume before diluting with pentanes. The previous step was repeated before collecting a fine-tan precipitate on a F porosity frit.

All cationic structures below represent triflate salts.

Representative Optimized Syntheses of Deuterated Cyclohexenes

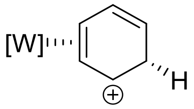

Synthesis of 2D

Used **General Procedure 1** with **1** (1.0404 g, 1.7904 mmol) in DCM (~5 mL) and HOTf (0.4007 g, 2.671 mmol) in MeOH (~2 mL). Yield: 1.0705 g (81.8%). Previously characterized. [3]

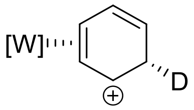

Synthesis of 6-*d_1*-2D

Used **General Procedure 1** with **1** (0.6112 g, 1.0512 mmol) in *d_2*-DCM (~3 mL) and DOTf (0.2729 g, 1.806 mmol) in MeOD (~2 mL). Yield: 0.6006 g (78.0%). Previously characterized. [3]

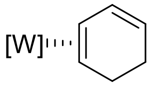

Synthesis of 3P

To 30-mL test tube was added **2D** (0.3916 g, 0.5356 mmol) and MeOH (~5 mL). The solution was chilled to -60 °C for five minutes before slowly adding $NaBH_4$ (0.1728 g, 4.567 mmol). After 30 minutes, the reaction mixture had changed from dark orange to tan. The reaction mixture was then diluted with DCM (5 mL) and washed with DI H_2O (3 x 5 mL). The organic layer was dried over anhydrous $MgSO_4$ and concentrated in vacuo. The resulting film was dissolved in minimal DCM and added to stirring pentanes (250 mL). Evaporated down to ~75 mL. Added 100 mL pentanes. Evaporated down to ~50 mL before repeating the previous step. A fine-tan precipitate was collected on a 30-mL M porosity frit and washed with 30 mL of pentanes (2 x 15 mL). Yield: 0.1611 g (51.6%). Previously characterized. [3]

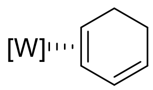

Synthesis of 3D

To 15-mL test tube was added **4D** (0.1886 g, 0.2572 mmol) and DCM (~3 mL). In a separate 15-mL test tube was added DCM (~1 mL) and DBU (0.1254 g, 0.8237 mmol). Both test tubes were chilled to -60 °C for 15 minutes before the solution of base was added to the solution of **4D**. After five minutes, the reaction mixture had changed from orange to tan. The reaction mixture was then diluted with DCM (5 mL) and washed with saturated NaOH (3 x 5 mL). The organic layer was dried over anhydrous Na_2SO_4 and evaporated to dryness. After confirming **3D** by ^{1}H NMR, the product was immediately used for next step. Previously characterized. [3]

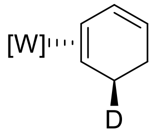

Synthesis of 5-*d₁*-3P

To 30-mL test tube was added **2D** (0.4980 g, 0.6811 mmol) and d_2-DCM (~3 mL). In a separate 30-mL test tube was added MeOD (~3 mL) and $NaBD_4$ (0.2411 g, 5.760 mmol). Both test tubes were chilled to -30 °C for 5 minutes before the solution of **2D** was added to the slurry of $NaBD_4$ in MeOD. After 30 minutes, the reaction mixture had changed from dark orange to tan. The reaction mixture was then diluted with DCM (5 mL) and washed with DI H_2O (3 x 5 mL). The organic layer was dried over anhydrous $MgSO_4$ and evaporated to dryness. After confirming **5-*exo*-d_1-3P** by ^{1}H NMR, the product was immediately used for next step.

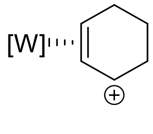

Synthesis of 4D

Synthesis and characterization have previously been reported. An alternate synthesis of **4D** is as follows: A 4-dram vial was charged with **7** (0.5442 g, 0.8905 mmol), 1,4 cyclohexadiene (2.7256 g, 34.01 mmol), and THF (3 mL). After stirring for 18 hours, the heterogeneous yellow reaction

mixture had become dark brown. The reaction mixture was loaded onto a silica column set in Et_2O. The tan-yellow band was collected and evaporated to an oil. A chilled (-30 °C) solution of HOTf (0.2632 g, 1.754 mmol) in DME (~2 mL) was added. Reaction mixture immediately turned dark red. Reaction mixture added to stirring Et_2O (250 mL) before collecting a tan precipitate on a 30-mL F frit and washing with 30 mL of Et_2O (2 x 15 mL). Yield: 0.3302 g (50.6%). Previously characterized. [3-4]

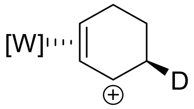

Synthesis of 6-*d₁*-4D

To 30-mL test tube was added **2D** (0.4980 g, 0.6811 mmol) and d_2-DCM (~3 mL). In a separate 30-mL test tube was added MeOD (~3 mL) and $NaBD_4$ (0.2411 g, 5.760 mmol). Both test tubes were chilled to -30 °C for 5 minutes before the solution of **2D** was added to the slurry of $NaBD_4$ in MeOD. After 30 minutes, the reaction mixture had changed from dark orange to tan. The reaction mixture was then diluted with DCM (5 mL) and washed with DI H_2O (3 x 5 mL). The organic layer was dried over anhydrous $MgSO_4$ and evaporated to dryness. A chilled (-30 °C) solution of HOTf (0.3188 g, 2.124 mmol) in DME (~3 mL) was added. Reaction mixture immediately turned dark red. Reaction mixture added to stirring Et_2O (300 mL) before collecting a tan precipitate on a 30-mL F frit and washing with 30 mL of Et_2O (2 x 15 mL). Yield: 0.3420 g (68.4%).

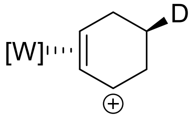

Synthesis of 5-*d₁*-4D

A 4-dram vial was charged with **6** (0.1262 g, 0.2164 mmol) and MeOD (~1 mL). A chilled (-60 °C) solution of d_2-DPhAT (0.1082 g, 0.3367 mmol) in MeOD (~1 mL) was added. Reaction mixture immediately turned dark red. After stirring for five minutes at room temperature, the reaction mixture was added to stirring Et_2O (300 mL). A tan precipitate was collected on a 30-mL F frit and washing with 15 mL of pentanes. Yield: 0.1263 g (79.5%).

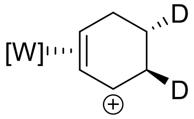

Synthesis of 4,5-*trans*-*d₂*-4D

To 30-mL test tube was added **6-*d₁*-2D** (0.3211 g, 0.4386 mmol) and MeOD (~5 mL). The solution was chilled to -60 °C for five minutes before slowly adding $NaBD_4$ (0.1341 g, 3.203 mmol). After one hour, the reaction mixture had changed from dark orange to tan. The reaction mixture was then diluted with DCM (5 mL) and washed with DI H_2O (2 x 5 mL). The organic layer was dried over anhydrous $MgSO_4$ and concentrated in vacuo. A chilled (-30 °C) solution of HOTf (0.3822 g, 2.547 mmol) in DME (~2 mL) was added. Reaction mixture immediately turned dark red. Reaction

mixture added to stirring Et_2O (250 mL) before collecting a tan precipitate on a 30-mL F frit and washing with 20 mL of Et_2O (2 x 10 mL). Yield: 0.1021 g (31.6%).

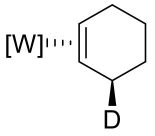

Synthesis of 3-*d₁*-5

Used **General Procedure 2** with **4D** (0.2858 g, 0.3898 mmol) in MeOD (~5 mL) and $NaBD_4$ (0.1501 g, 2.671 mmol). Yield: 0.0751 g (32.9%).

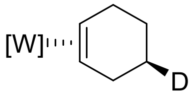

Synthesis of 4-*d₁*-5

Used **General Procedure 2** with **6-*d₁*-4D** (0.3405 g, 0.4638 mmol) in MeOH (~5 mL) and $NaBH_4$ (0.2139 g, 5.653 mmol). Yield: 0.1445 g (53.2%).

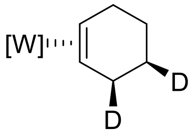

Synthesis of *cis*-3,4-*d₂*-5

Used **General Procedure 2** with **6-*d₁*-4D** (0.3655 g, 0.4978 mmol) in MeOD (~5 mL) and $NaBD_4$ (0.3485 g, 8.325 mmol). Yield: 0.1533 g (52.4%).

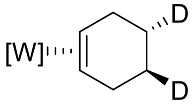

Synthesis of *trans*-4,5-*d₂*-5

Used **General Procedure 2** with **4,5-*trans*-*d₂*-4D** (0.1021 g, 0.1389 mmol) in MeOH (~3 mL) and $NaBH_4$ (0.0581 g, 1.535 mmol). Yield: 0.0722 g (88.5%).

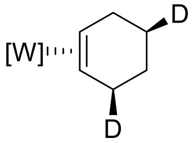

Synthesis of *cis*-3,5-*d₂*-5

Used **General Procedure 2** with **5-*d₁*-4D** (0.1198 g, 0.1632 mmol) in MeOD (~5 mL) and $NaBD_4$ (0.0871 g, 2.08 mmol). Yield: 0.0481 g (50.2%).

NMR Spectra

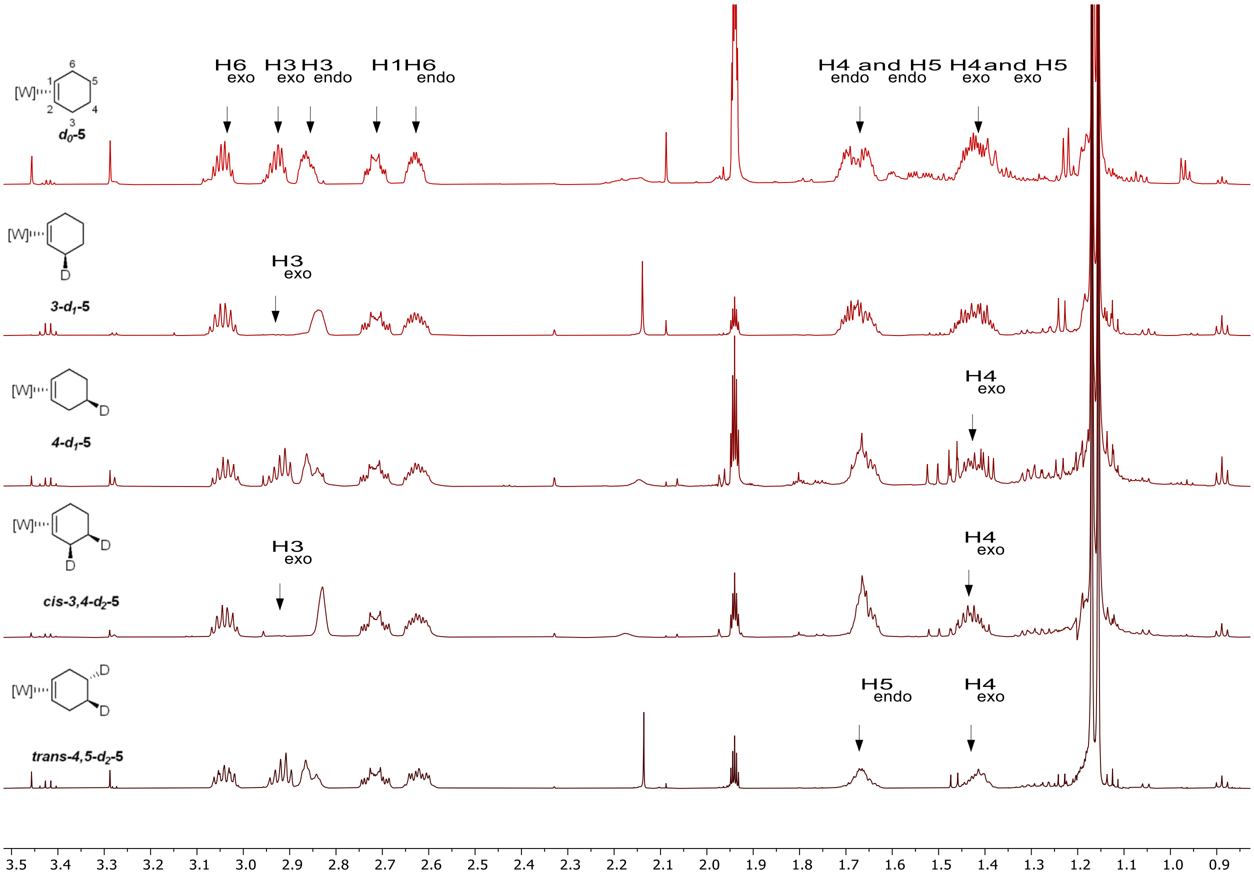

Figure S1: ^{1}H NMR Comparison of ***d$_0$*-5** (800 MHz, d_4-MeCN, 25 °C, top)., **3-*d$_1$*-5**, **4-*d$_1$*-5**, ***cis*-3,4-*d$_2$*-5**, and ***trans*-4,5-*d$_2$*-5** (600 MHz, d_4-MeCN, 25 °C).

Supporting Information on Molecular Rotational Spectroscopy Analysis

Contents:

A) New Sample System for Thermolysis of Tungsten Complexes

B) Isotopologue and Isotopomer Analysis of Deuterated Cyclohexenes

C) Chiral Analysis of d_1-Cyclohexenes Prepared with Enantioenriched Tungsten Complexes

Data Availability:

All molecular rotational resonance (MRR) spectra acquired for sample analysis in this work can be accessed from Zenodo:

https://doi.org/10.5281/zenodo.15548822

This data archive also includes the full spectroscopic fit results including centrifugal distortion constants when needed to achieve a good fit. The fit results in the repository also contain the list of assigned transitions with the fit summaries of each species. In this SI section, the fit results only report the rotational constants which are the spectroscopic quantities used to identify molecular geometries. The Zenodo repository includes the data used to calculate the enantiomeric excess of enantioenriched cyclohexene-d_1 samples prepared with enantioenriched tungsten complexes.

A) New Sample System for Thermolysis of Tungsten Complexes

As discussed in the manuscript, the method used in the previous work to remove the cyclohexene ligand from the tungsten complex by heating used long heating times to liberate enough sample for MRR analysis.[1] There was strong evidence that these long heating times scrambled and modified the cyclohexene deuteration patterns that were imprinted in the initial synthesis. For this work, a new sample system was designed to allow rapid, high-temperature heating with the goal of liberating the cyclohexene on a time scale that is fast compared to the competing on-metal isomerization and hydrogen substitution reactions. As shown in Figure 2 of the manuscript, the new sample system gives cyclohexene gas samples with fewer and lower impurities. Furthermore, the sample composition is stable over multiple heating cycles of the same sample loading.

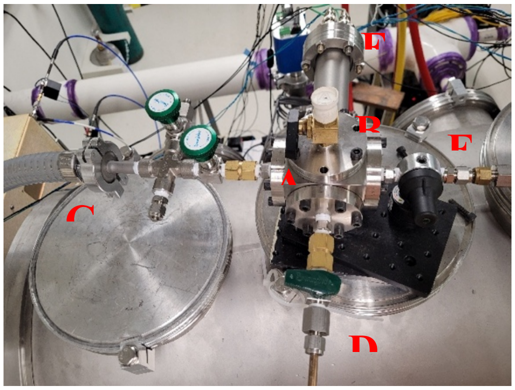

Figure S2: The thermolysis system used to release cyclohexene from the tungsten metal complex by thermal dissociation is shown. The main sample cell, A, is a stainless-steel cube where the sample is loaded through the top flange, B. The cell can be evacuated using a dry scroll pump through the vacuum line, C. Heating occurs under a neon atmosphere where neon is introduced through the valve D. The electrical connections for the heating crucible are on a side tube, E. The final gas mixture is sent to the spectrometer through a fixed pressure regulator, F, with outlet pressure 15 psig.

An image of the new sample system is shown in Figure S2. The main sample chamber is a stainless-steel cube with 2.75” conflat flanges (A). Sample is placed in the heating crucible, Figure S3, by removing the top flange, (B). The top flange also has a basic septum port above a ball valve. This port is used to inject liquid propylene oxide into the sample chamber for chiral tag measurements. The sample chamber is connected to a dry scroll pump, (C). The neon gas used for rotational spectroscopy pulsed-jet measurements can be introduced through the side valve, (D). The output of the sample cell passes through a fixed regulator, F, with output pressure of 15 psig, and goes to the sample inlet on the chirped-pulse FTMW spectrometer.[5-6] There is a side stainless tube, E, that has the electrical connections for the cartridge heater and thermocouple used for temperature control.

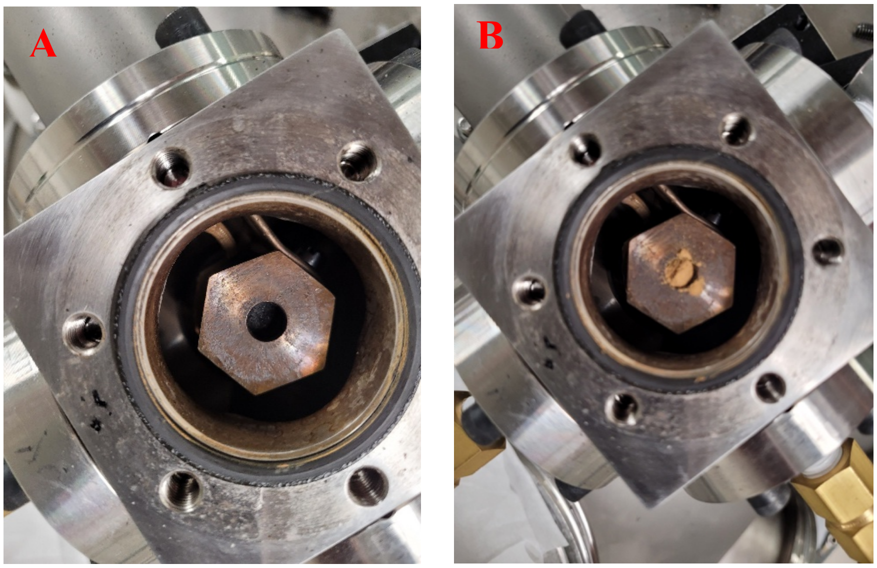

Figure S3: The stainless-steel sample holder is shown. The sample holder has a cartridge heater and thermocouple used to control the temperature. The sample holder is mounted on a Vespel post and has no direct thermal contact to the sample cell walls. The sample can be loaded directly as a solid powder as shown in (B). However, the more common method of loading the sample is to dissolve it in 100 μL of DCM and transfer the solution to the holder.

The stainless-steel heating crucible for the sample is shown in Figure S3. The crucible is thermally isolated from the rest of the cell using a Vespel support post. This material was chosen for its very low thermal conductivity and high temperature working limit. A cartridge heater and thermocouple are attached to the sample-holding crucible for heating samples with a temperature controller (the thermocouple attachment is visible to the top right and the wrapped connector to the cartridge heater is also visible). Figure S3B shows the crucible loaded with about 40 mg of tungsten metal complex.

Measurement Method

The tungsten metal complex is loaded into the crucible in one of two ways. As shown in Figure S3B, the solid powder can be loaded directly when the sample amount is large. However, the more commonly used method for loading the sample was to first dissolve the metal complex in 100 μL of dichloromethane (DCM) and then transfer the solution to the crucible. This method was required for small samples where the metal complex was mainly present as a film on the walls of a glass sample vial.

After transferring the sample to the crucible, the sample cell is closed and connected briefly to the dry scroll pump to remove the air. The sample cell is then refilled to 1 atm of neon. The sample crucible temperature is increased to 100ºC and held there for 10 min to fully evaporate the (DCM) and release other volatiles. The sample chamber is again opened to the dry scroll pump until the pressure at the pump inlet reaches its limit of about 20 mTorr. After evacuating the volatiles, neon is added to 1atm for the subsequent heating cycle.

To release the cyclohexene. The sample holder is heated to 250ºC (523K) and held at this temperature for 5 min. As shown in Figure 2 of the main paper, this heating cycle is expected to release cyclohexene from more than 95% of the sample. It takes about 5 minutes for the sample crucible to reach the final temperature. After holding the temperature at 250ºC for 5 minutes, the crucible heater is turned off. The sample holder cools to 120ºC in about 5 minutes. At this point, 17 μL of propylene oxide (TCI America P3117 EE > 97%) is added to the sample cell through the septum port if the measurement is a chiral analysis. This amount of chiral tag sample makes a 0.4% mixture in neon in the analyses. Finally, neon is added to the sample cell at a crucible temperature of 120ºC for a final total cell pressure of 4 atm. After waiting a short time for gas mixing, the spectrum acquisition begins. It is important to note that the sample holder is thermally isolated from the stainless-steel sample cell. During this sampling process, the walls of the sample cell remain near room-temperature and are never perceptibly warm to the touch. As a result, any cyclohexene liberated from the metal complex is expected to rapidly cool to near room-temperature in the neon atmosphere of the sample cell. When the measurement is complete, the sample holder is heated to 250ºC under vacuum from the dry scroll pump for 10 minutes to prepare the sample system for the next measurement.

B) Isotopologue and Isotopomer Analysis of Deuterated Cyclohexenes

The use of molecular rotational resonance (MRR) spectroscopy to analyze the isotopic composition of deuterated cyclohexene released from the tungsten metal complex by thermolysis has been described in detail in the supporting information section of the previous work.[1] A summary of the spectroscopy apporach is provided here.

Under the Born-Oppenheimer approximation, the equilibrium geometry is the same for all isotopic variants of the molecule. Therefore, a single geometry of cyclohexene can be used to predict the rotational constants, inversely proportional to the moments-of-inertia calculated in the principal axis system of overall molecular rotation, by simply changing the nuclear mass for each deuterium substituted atom position.[7] Cyclohexene with specific deuteration patterns in the experimental sample are identified by first assigning the rotational spectrum through the identification of several allowed transitions between the quantized energy levels of the kinetic rotational energy.[8] This analysis provides experimental rotational constants. The experimental rotational constants are then compared to the predicted rotational constants of all possible deuteration patterns of cyclohexene calculated using a single reference geometry. A specific deuterated cyclohexene structure is attributed to an experimental spectrum when the root-mean-squared percent error for the three rotational constants (A, B, C) is small – less than about 0.02% error – and the next best matching deuteration pattern has a significantly larger error.

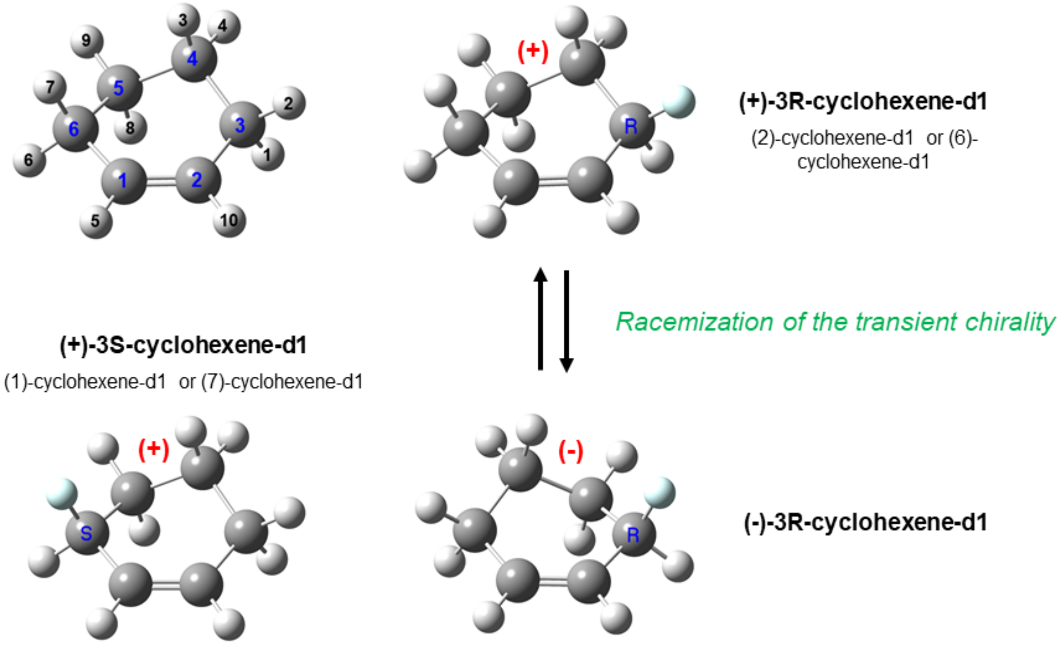

Figure S4: The equilibrium geometry of cyclohexene is shown to the top left with the atom numbering system used in this SI document. The ring pucker of cyclohexene is an element of transient chirality that is equilibrated in the gas sample. When deuteration creates a new chiral center, two diastereomer spectra are observed as shown by the two structures to the right.

The reference structure for cyclohexene and atom labeling convention is shown at the top left of Figure S4. The carbon atom labels used to identify the deuteration positions in the main paper are shown in blue. The hydrogen atom labels used to specify the deuterium labeling pattern for the assigned MRR spectra are shown in black. One complication in the rotational spectroscopy of cyclohexene is that the ring pucker gives rise to conformational enantiomers. However, these enantiomers can interconvert through low barrier conformational isomerization and can only be isolated transiently – a behavior known as transient chirality in the field of rotational spectroscopy.[9] In rotational spectroscopy measurements, there will be rapid racemization of the conformational enantiomers in the near room-temperature gas mixture prior to sample injection into the spectrometer.

To account for the effects of the conformational chirality on the rotational spectroscopy of deuterated cyclohexene, the conformational enantiomer is identified by the slope of the bond between the carbon atoms opposite the alkene double bond as shown in Figure S4. The slope can either be positive (+) or negative (-) in the two enantiomers for the ring-puckering in cyclohexane-d_0. Therefore, in cases where deuteration creates an isotopically chiral center, there will be two diastereomers present in the measurement at equal abundance. This structural feature is illustrated by considering the (+)-3R-cyclohexene-d1 enantiomer in top right of the figure, where name is based on the carbon atom deuteration site as used in the main paper. By comparison to the figure in the top left that gives the full labeling convention, this structure is

designated (2)-cyclohexene-d1 when using the hydrogen atom labels. By the C_2-symmetry of the cyclohexene reference geometry, this structure is equivalent to (6)-cyclohexene-d1 (H-atom label). However, the sample will include an equal amount of deuterated cyclohexene where the ring-pucker isomers have equilibrated so that (-)-3R-cyclohexene-d1 is expected in equal abundance. Enantiomers have identical mass distributions and, therefore, rotational constants. As a result, the rotational constants of (-)-3R-cyclohexene-d1are equivalent to its enantiomer, (+)-3S-cyclohexene-d1 shown in the bottom left. Using the reference geometry at the top left, this species is (7)-cyclohexene-d1 (equivalent to (1)-cyclohexene-d1 by C_2-symmetry) and two distinct MRR spectra are observed when (+)-3R-cyclohexene-d1 is released from the tungsten complex.

A summary of all deuterated cyclohexenes that have been identified in the previous and current study is presented in Table S1. This table identifies the deuteration pattern using the hydrogen atom labels in the reference geometry shown in the top left of Figure S4. This table is an update from the table reported in the previous SI section. There are four changes (highlighted in red in Table S1):

1) The previous table swapped the identifications of (4)-d_1 and d(5)-d_1 and these are now corrected.

2) There was an error in reporting the C rotational constant for the (2,3,8)-d_3 deuteration pattern that has been corrected.

3) Two new species have been identified in the current set of samples – (1,3,6)-d_2 and (2,4,7)-d_2 – and these results are now included in the summary table.

Table S1. Comparison between calculated and experimental rotational constants of deuterium isotopologues of cyclohexene. The theoretical rotational constants are scaled using the refined structure determined from the isotopologues observed in natural abundance. Experimental fits were done using Pickett's SPCAT/SPFIT analysis program.

	Theoretical Rotational Constants			Experimental Rotational Constants			RMS error (%)	RMS next closest (%)
	A (MHz)	B (MHz)	C (MHz)	A (MHz)	B (MHz)	C (MHz)		
(1)-d1	4684.219	4358.111	2517.572	4684.2226 (0.00008%)	4358.123 (0.0003%)	2517.5716 (-0.000016%)	0.0004	0.9989, 1.1854
(2)-d1	4728.048	4314.878	2490.756	4728.0271 (-0.0004%)	4314.894 (0.0004%)	2490.7470 (-0.0004%)	0.0004	1.0037, 1.7424
(3)-d1	4598.989	4448.217	2540.135	4598.9903 (0.00003%)	4448.224 (0.00016%)	2540.1285 (-0.0003%)	0.0005	1.3708, 1.4541
(4)-d1	4628.066	4407.568	2486.842	4628.0530 (-0.0003%)	4407.556 (-0.0003%)	2486.8528 (0.0004%)	0.0004	0.1626, 1.1914
(5)-d1	4618.901	4402.491	2482.75	4618.9129 (0.0003%)	4402.508 (0.0004%)	2482.7260 (-0.0010%)	0.0011	0.163, 1.2893
(4,9)-d2	4421.288	4375.256	2416.333	4421.811 (0.012%)	4375.1906 (-0.0015%)	2416.4423 (0.005%)	0.0076	0.3695, 0.485
(3,8)-d2	4456.721	4360.738	2519.087	4456.8531 (0.003%)	4360.890 (0.003%)	2519.1458 (0.002%)	0.003	1.312, 1.4409
(4,8)-d2	4480.6184	4330.3479	2466.3685	4480.8460 (0.005%)	4330.5911 (0.006%)	2466.4428 (0.003%)	0.0047	0.2001, 0.9340
(2,4)-d2	4562.684	4239.535	2419.902	4562.8274 (0.003%)	4239.7120 (0.004%)	2419.9967 (0.004%)	0.004	0.1973, 0.2273
(1,3)-d2	4542.793	4269.867	2496.803	4543.0663 (0.006%)	4269.946 (0.0019%)	2496.8638 (0.002%)	0.0043	0.0562, 0.7313
(2,3)-d2	4584.407	4233.947	2469.753	4584.6894 (0.006%)	4233.998 (0.0012%)	2469.8934 (0.006%)	0.0047	0.1083, 0.8206
(1,4)-d2	4546.732	4258.462	2445.308	4547.2620 (0.012%)	4258.508 (0.0011%)	2445.4059 (0.004%)	0.0095	0.1763, 0.2178
(1,6)-d2	4671.5817	4143.0032	2447.6479	4671.9466 (0.008%)	4143.1205 (0.003%)	2447.6730 (0.0010%)	0.0048	0.1188, 0.9342
(2,9)-d2	4581.0412	4218.6573	2418.2439	4581.0458 (0.00010%)	4219.0130 (0.008%)	2418.3460 (0.004%)	0.0054	0.2548, 0.0042
(1,8)-d2	4542.8417	4273.9475	2496.0786	4543.0236 (0.004%)	4274.228 (0.007%)	2496.1954 (0.005%)	0.0052	0.0606, 0.7041
(1,9)-d2	4535.321	4265.041	2443.964	4535.320 (-0.00002%)	4265.422 (0.009%)	2444.0443 (0.003%)	0.0055	0.1757, 0.2145
(2,8)-d2	4587.667	4226.731	2468.865	4587.8261 (0.003%)	4226.954 (0.005%)	2468.9146 (0.002%)	0.0038	0.1066, 0.8276
(1,5)-d2	4536.012	4251.504	2439.721	4536.705 (0.015%)	4251.294 (-0.005%)	2439.7907 (0.003%)	0.0094	0.2069, 0.2119

(2,5)-d2	4549.062	4234.304	2414.656	4549.7472 (0.015%)	4234.144(-0.004%)	2414.7016 (0.0019%)	0.0090	0.2005, 0.2187
(1,10)-d2	4519.2719	4270.5929	2441.0246	4519.399 (0.003%)	4270.658 (-0.0015%)	2441.122 (0.004%)	0.003	0.2273,0.3364
(2,10)-d2	4563.1005	4226.7668	2416.2462	4563.434 (0.007%)	4226.355 (-0.010%)	2416.498 (0.010%)	0.0093	0.1978, 0.2163
(2,3,8)-d3	4448.28	4148.446	2449.104	4448.655 (0.008%)	4148.763 (0.008%)	2449.263 (0.0065%)	0.0075	0.5691, 0.6681
(1,4,9)-d3	4328.111	4244.478	2376.592	4328.3962 (0.007%)	4245.123 (0.015%)	2376.7715 (0.008%)	0.0108	0.3581, 0.3627
(2,4,9)-d3	4405.188	4188.755	2476.620	4405.5468 (0.008%)	4189.116 (0.009%)	2476.7582 (0.006%)	0.0076	0.7374, 0.8750
(1,3,8)-d3	4366.493	4202.289	2352.164	4366.570 (0.0018%)	4202.971 (0.016%)	2352.3619 (0.008%)	0.0106	0.3785, 0.3787
(1,3,6)-d3	4531.28	4061.96	2427.44	4531.458 (0.0038%)	4061.846 (0.0028%)	2427.386 (0.0023%)	0.0030	0.1178, 0.1440
(2,4,7)-d3	4499.25	4081.47	2378.78	4499.436 (0.0042%)	4081.292 (0.0044%)	2378.800 (0.0008%)	0.0036	0.1071, 0.1241
(2,3,7,8)-d4	4395.524	3989.456	2408.355	4396.2300 (0.016%)	3989.815 (0.009%)	2408.5446 (0.008%)	0.0116	0.1099, 0.8497
(2,4,7,9)-d4	4317.699	4039.583	2313.929	4318.2255 (0.012%)	4040.268 (0.017%)	2314.1446 (0.009%)	0.0135	0.1087, 0.2349

The composition analysis of deuterated cyclohexene samples prepared by thermolysis of the tungsten complex quantifies the amount of all d_0-d_3 species that have been identified (Table S1). No samples in the present work had the d_4-spectra in Table S1 present, so this species is omitted from the sample composition reports. The relative amount of each deuterated cyclohexene is quantified by the signal of the highest intensity rotational spectroscopy transition in the measured 5.5 – 7.5 GHz frequency range. This transition correlates to the $1_{11} – 0_{00}$ transition of cyclohexene-*d0* at 7301.5625 MHz. Representative spectra of the isotopologues and isotopomers prepared in this study are shown in Figure S5. The samples with the highest fractional abundance of the target deuterated cyclohexene are chosen for this figure. The transition frequencies corresponding to each chemically distinct species – listed using the carbon atom labels reported in the manuscript – are listed in Table S2. As discussed above, deuteration patterns that create new chiral centers – unless the structure is *meso* – are present in two diastereomers caused by the ring-pucker. The sum of the transitions from the two diastereomers is used to quantify the sample composition. A further complication is that some spectra show resolved nuclear quadrupole hyperfine structure from the *I=1* deuterium nucleus.[8] This hyperfine splitting can produce two or three observed transition peaks – dominated by the central component. In cases where the hyperfine structure is observed, the sum of the set of peaks is used to quantify the amount of the species present in the sample. Finally, when converting spectral intensities to relative abundances, the observed transition intensities need to be corrected by the square of the transition dipole moment.[8] These vary between isotopic species due to rotation of the principal axis system associated with the change in the mass distribution caused by deuteration. These changes can be calculated from the reference geometry and dipole moment vector by rotation of the axis system. The rotated dipole moment components for the transitions used to determine the sample composition are also reported in the Table S2.

Table S2: The frequencies of the transition peaks, including hyperfine components in some cases (Freq 1 – Freq 3 – as required), used to quantify the sample composition of deuterated cyclohexene samples are given. The species are designated by the carbon atom labels of Figure S4. The dipole moment component for the observed transition is also reported.

Deuterated Species	Ring Pucker Isomer 1			Dipole Moment (Debye)	Ring Pucker Isomer 2			Dipole Moment (Debye)
	Freq 1 (MHz)	Freq 2 (MHz)	Freq 3 (MHz)		Freq 1 (MHz)	Freq 2 (MHz)	Freq 3 (MHz)	
d0	7301.5625			0.330				
1-d1	7101.6500			0.256				
3-d1	7201.8000	7201.9000		0.330	7218.7750	7218.8750		0.330
4-d1	7139.1375	7139.2250		0.324	7114.9125			0.266
cis-3,4-d2	6992.6750	6992.7750		0.302	7054.5375	7054.6125	7054.6875	0.330
trans-3,4-d2	7039.8875	7039.9750	7040.0500	0.328	6982.8270			0.317
cis-3,6-d2	7119.5750	7119.6500	7119.7375	0.330				
cis-4,5-d2	6947.2500	6947.3250		0.251				
trans-4,5-d2	6975.9500	6976.0375		0.330	6791.6125	6791.7000		0.330
cis-3,5-d2	7039.1750	7039.2500	7039.3250	0.329	6999.4000	6999.4880		0.311
trans-3,5-d2	6979.3375			0.307	7056.7000	7056.7875	7056.8625	0.328
1,3-d2	6976.4875			0.297	6964.4625			0.315
2,3-d2	6960.5413			0.304	6979.9469			0.309
cis-3,4-trans-5-d3	6705.1500	6705.2875		0.329	6897.8625	6897.9500		0.330
trans-3,4-trans-6-d3	6878.1875	6878.2625	6878.3375	0.328	6958.8000	6958.8875		0.325
trans-3,4-trans-5-d3	6882.2833			0.330	6718.8997			0.328

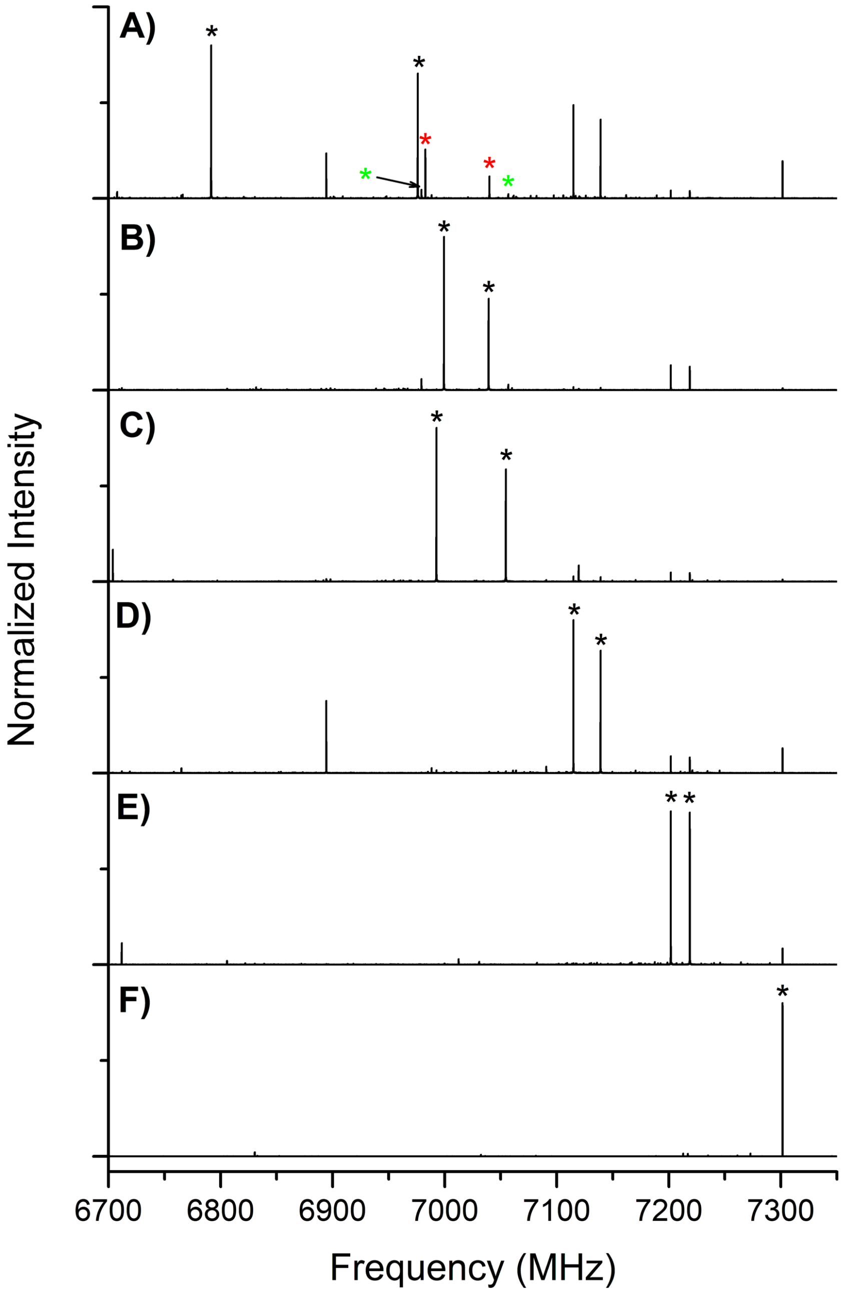

Figure S5: Representative MRR spectra of the five deuterated cyclohexenes prepared for this study are shown. (A) trans-4,5-d2 (Figure 3 Trial 1), (B) cis-3,5-d2 (Figure4 Trial 3), (C) cis-3,4-d2 (Figure 3 Trial 3), (D) 4-d1 (Figure 3 Trial 1), (E) 3-d1 (Figure 3 Trial 3), (E) d0 (commercial sample). In cases where several syntheses were performed for the same target, the sample with the highest abundance of the target species is shown. These spectra are normalized to the highest intensity rotational transition. The black asterisks identify the rotational transitions that are related to the 1_{11}-0_{00} transition of cyclohexene-d0 (Panel (F)). All of the deuterated cyclohexenes are chiral so that transitions from two ring pucker isomers are observed instead of the single transition for cyclohexene-d0. As an illustration of the composition analysis, consider the spectrum of trans-4,5-d2 in Panel (A). The two transitions in red identify a significant impurity of the trans-3,4-d2 isotopomer (10.7%). As can be seen from the spectrum comparison, the trans-4,5-d2 sample also has a significant amount of 4-d1 (26.5%) and d0 (4.2%) impurities. A lower amount of 3-d1 (2.0%) impurity is also evident in the spectrum. The green asterisks identify the two transitions from the ring pucker isomers of the trans-3,5-d2 isotopomer impurity (2.1%).

The sample composition is reported in two ways. First, the relative fractional abundance of each detected species, referenced to the highest abundance component, is given. These results are reported in the main manuscript. The percent composition of the sample is also calculated using the total amount of detected deuterated cyclohexene species. The sample composition reports for the analyses reported in the manuscript are given on the following pages. They are presented in the order that the sample is reported in the manuscript:

Table S3. Summary of MRR data for cyclohexenes.

Target Species	3d1	
Figure	3	
Trial	1	
Isotopic Species	Fraction of Dominant	Percentage Composition
3-d1	1.00	93.4
d0	0.06	5.4
4-d1	0.01	0.8
1-d1	0.00	0.4
cis-3,4-d2	0	0
trans-3,4-d2	0	0
cis-3,6-d2	0	0
cis-4,5-d2	0	0
trans-4,5-d2	0	0
cis-3,5-d2	0	0
trans-3,5-d2	0	0
1,3-d2	0	0
2,3-d2	0	0
cis-3,4-trans-5-d3	0	0
trans-3,4-trans-6-d3	0	0
trans-3,4-trans-5-d3	0	0

Target Species	3d1	
Figure	3	
Trial	2	
Isotopic Species	Fraction of Dominant	Percentage Composition
3-d1	1.00	94.0
d0	0.05	4.5
4-d1	0.01	1.0
1-d1	0.01	0.5
cis-3,4-d2	0	0
trans-3,4-d2	0	0
cis-3,6-d2	0	0
cis-4,5-d2	0	0
trans-4,5-d2	0	0
cis-3,5-d2	0	0
trans-3,5-d2	0	0
1,3-d2	0	0
2,3-d2	0	0
cis-3,4-trans-5-d3	0	0
trans-3,4-trans-6-d3	0	0
trans-3,4-trans-5-d3	0	0

Target Species	3d1	
Figure	3	
Trial	3	
Isotopic Species	Fraction of Dominant	Percentage Composition
3-d1	1.00	94.0
d0	0.06	5.5
4-d1	0.01	0.5
1-d1	0	0
cis-3,4-d2	0	0
trans-3,4-d2	0	0
cis-3,6-d2	0	0
cis-4,5-d2	0	0
trans-4,5-d2	0	0
cis-3,5-d2	0	0
trans-3,5-d2	0	0
1,3-d2	0	0
2,3-d2	0	0
cis-3,4-trans-5-d3	0	0
trans-3,4-trans-6-d3	0	0
trans-3,4-trans-5-d3	0	0

Target Species	4d1	
Figure	3	
Trial	1	
Isotopic Species	Fraction of Dominant	Percentage Composition
4-d1	1.000	83.2
3-d1	0.096	8.0
d0	0.065	5.4
cis-3,4-d2	0.018	1.5
cis-3,5-d2	0.007	0.6
1-d1	0.005	0.4
cis-3,6-d2	0.005	0.4
trans-3,5-d2	0.002	0.2
trans-4,5-d2	0.001	0.1
trans-3,4-d2	0.001	0.1
cis-4,5-d2	0	0
1,3-d2	0	0
2,3-d2	0	0
cis-3,4-trans-5-d3	0	0
trans-3,4-trans-6-d3	0	0
trans-3,4-trans-5-d3	0	0

Target Species	4d1	
Figure	3	
Trial	2	
Isotopic Species	Fraction of Dominant	Percentage Composition
4-d1	1.000	81.3
3-d1	0.086	7.0
d0	0.064	5.2
cis-3,4-d2	0.048	3.9
cis-3,5-d2	0.016	1.3
cis-3,6-d2	0.006	0.5
2,3-d2	0.004	0.3
1-d1	0.004	0.3
trans-3,4-d2	0.003	0.2
cis-4,5-d2	0	0
trans-4,5-d2	0	0
1,3-d2	0	0
trans-3,5-d2	0	0
cis-3,4-trans-5-d3	0	0
trans-3,4-trans-6-d3	0	0
trans-3,4-trans-5-d3	0	0

Target Species	4d1	
Figure	3	
Trial	3	
Isotopic Species	Fraction of Dominant	Percentage Composition
4-d1	1.000	82.9
3-d1	0.077	6.4
d0	0.063	5.2
cis-3,4-d2	0.048	4.0
cis-3,5-d2	0.011	0.9
cis-3,6-d2	0.005	0.4
1-d1	0.003	0.3
cis-4,5-d2	0	0
trans-4,5-d2	0	0
trans-3,4-d2	0	0
trans-3,5-d2	0	0
1,3-d2	0	0
2,3-d2	0	0
cis-3,4-trans-5-d3	0	0
trans-3,4-trans-6-d3	0	0
trans-3,4-trans-5-d3	0	0

Target Species	4d1	
Figure	3	
Trial	4	
Isotopic Species	Fraction of Dominant	Percentage Composition
4-d1	1.000	85.2
3-d1	0.080	6.8
d0	0.067	5.7
cis-3,4-d2	0.027	2.3
1-d1	0	0
trans-3,4-d2	0	0
cis-3,6-d2	0	0
cis-4,5-d2	0	0
trans-4,5-d2	0	0
cis-3,5-d2	0	0
trans-3,5-d2	0	0
1,3-d2	0	0
2,3-d2	0	0
cis-3,4-trans-5-d3	0	0
trans-3,4-trans-6-d3	0	0
trans-3,4-trans-5-d3	0	0

Target Species	4d1	
Figure	3	
Trial	5	
Run	1	
Isotopic Species	Fraction of Dominant	Percentage Composition
4-d1	1.000	88.0
3-d1	0.085	7.5
d0	0.051	4.5
1-d1	0	0
cis-3,4-d2	0	0
trans-3,4-d2	0	0
cis-3,6-d2	0	0
cis-4,5-d2	0	0
trans-4,5-d2	0	0
cis-3,5-d2	0	0
trans-3,5-d2	0	0
1,3-d2	0	0
2,3-d2	0	0
cis-3,4-trans-5-d3	0	0
trans-3,4-trans-6-d3	0	0
trans-3,4-trans-5-d3	0	0

Target Species	4d1	
Figure	3	
Trial	5	
Run	2	
Isotopic Species	Fraction of Dominant	Percentage Composition
4-d1	1.000	86.2
3-d1	0.098	8.4
d0	0.053	4.5
cis-3,6-d2	0.006	0.5
trans-4,5-d2	0.003	0.3
cis-3,4-d2	0	0
trans-3,4-d2	0	0
cis-4,5-d2	0	0
1-d1	0	0
cis-3,5-d2	0	0
trans-3,5-d2	0	0
1,3-d2	0	0
2,3-d2	0	0
cis-3,4-trans-5-d3	0	0
trans-3,4-trans-6-d3	0	0
trans-3,4-trans-5-d3	0	0

Target Species	4d1	
Figure	3	
Trial	5	
Run	3	
Isotopic Species	Fraction of Dominant	Percentage Composition
4-d1	1.000	85.5
3-d1	0.097	8.3
d0	0.049	4.2
cis-3,5-d2	0.015	1.3
1-d1	0.005	0.4
1,3-d2	0.003	0.3
trans-3,4-d2	0	0
cis-3,6-d2	0	0
trans-4,5-d2	0	0
cis-3,4-d2	0	0
trans-3,5-d2	0	0
cis-4,5-d2	0	0
2,3-d2	0	0
cis-3,4-trans-5-d3	0	0
trans-3,4-trans-6-d3	0	0
trans-3,4-trans-5-d3	0	0

Target Species	Cis-3,4-d2	
Figure	3	
Trial	1	
Isotopic Species	Fraction of Dominant	Percentage Composition
cis-3,4-d2	1.000	68.7
3-d1	0.297	20.4
cis-3,6-d2	0.054	3.7
4-d1	0.050	3.4
d0	0.026	1.8
cis-4,5-d2	0.010	0.7
trans-3,4-trans-6-d3	0.009	0.6
2,3-d2	0.007	0.5
1-d1	0.004	0.3
cis-3,5-d2	0	0
trans-3,4-d2	0	0
1,3-d2	0	0
trans-3,5-d2	0	0
cis-3,4-trans-5-d3	0	0
trans-4,5-d2	0	0
trans-3,4-trans-5-d3	0	0

Target Species	Cis-3,4-d2	
Figure	3	
Trial	2	
Isotopic Species	Fraction of Dominant	Percentage Composition
cis-3,4-d2	1.000	59.2
cis-3,4-trans-5-d3	0.424	25.1
trans-3,4-trans-6-d3	0.088	5.2
3-d1	0.042	2.5
4-d1	0.036	2.1
trans-3,5-d2	0.026	1.6
cis-3,6-d2	0.020	1.2
trans-4,5-d2	0.020	1.2
cis-4,5-d2	0.010	0.6
d0	0.009	0.5
2,3-d2	0.008	0.5
trans-3,4-d2	0.004	0.2
cis-3,5-d2	0.002	0.1
1,3-d2	0	0
1-d1	0	0
trans-3,4-trans-5-d3	0	0

Target Species	Cis-3,4-d2	
Figure	3	
Trial	3	
Isotopic Species	Fraction of Dominant	Percentage Composition
cis-3,4-d2	1.000	84.6
cis-3,6-d2	0.076	6.4
3-d1	0.048	4.1
4-d1	0.033	2.8
cis-4,5-d2	0.008	0.7
2,3-d2	0.007	0.6
d0	0.005	0.4
trans-3,4-trans-6-d3	0.004	0.4
cis-3,4-trans-5-d3	0.002	0.2
trans-3,4-d2	0	0
trans-3,5-d2	0	0
1,3-d2	0	0
1-d1	0	0
cis-3,5-d2	0	0
trans-4,5-d2	0	0
trans-3,4-trans-5-d3	0	0

Target Species	trans-4,5-d2	
Figure	3	
Trial	1	
Isotopic Species	Fraction of Dominant	Percentage Composition
trans-4,5-d2	1.000	52.4
4-d1	0.506	26.5
trans-3,4-d2	0.203	10.7
d0	0.080	4.2
trans-3,5-d2	0.041	2.1
3-d1	0.038	2.0
cis-4,5-d2	0.011	0.6
cis-3,4-trans-5-d3	0.008	0.4
trans-3,4-trans-6-d3	0.004	0.2
cis-3,4-d2	0.004	0.2
1,3-d2	0.004	0.2
1-d1	0.002	0.1
trans-3,4-trans-5-d3	0.002	0.1
cis-3,5-d2	0.002	0.1
cis-3,6-d2	0.002	0.1
2,3-d2	0	0

Target Species	trans-4,5-d2	
Figure	3	
Trial	2	
Isotopic Species	Fraction of Dominant	Percentage Composition
4-d1	1.000	66.1
trans-4,5-d2	0.188	12.4
d0	0.120	8.0
3-d1	0.085	5.6
trans-3,4-d2	0.055	3.7
trans-3,5-d2	0.033	2.2
cis-4,5-d2	0.017	1.2
1-d1	0.009	0.6
cis-3,5-d2	0.006	0.4
cis-3,6-d2	0	0
cis-3,4-d2	0	0
1,3-d2	0	0
2,3-d2	0	0
cis-3,4-trans-5-d3	0	0
trans-3,4-trans-6-d3	0	0
trans-3,4-trans-5-d3	0	0

Target Species	Enantioenriched (R)-4d1	
Figure	4	
Trial	1	
Isotopic Species	Fraction of Dominant	Percentage Composition
4-d1	1.000	70.6
d0	0.319	22.5
3-d1	0.097	6.9
1-d1	0	0
cis-3,4-d2	0	0
trans-3,4-d2	0	0
cis-3,6-d2	0	0
cis-4,5-d2	0	0
trans-4,5-d2	0	0
cis-3,5-d2	0	0
trans-3,5-d2	0	0
1,3-d2	0	0
2,3-d2	0	0
cis-3,4-trans-5-d3	0	0
trans-3,4-trans-6-d3	0	0
trans-3,4-trans-5-d3	0	0

Target Species	Cis-3,5-d2	
Figure	4	
Trial	1	
Isotopic Species	Fraction of Dominant	Percentage Composition
3-d1	1.000	48.0
cis-3,5-d2	0.954	45.8
4-d1	0.029	1.4
d0	0.028	1.4
1,3-d2	0.021	1.0
cis-3,4-d2	0.018	0.9
trans-3,5-d2	0.012	0.6
1-d1	0.008	0.4
cis-3,6-d2	0.007	0.4
trans-3,4-d2	0.007	0.3
cis-4,5-d2	0	0
trans-4,5-d2	0	0
2,3-d2	0	0
cis-3,4-trans-5-d3	0	0
trans-3,4-trans-6-d3	0	0
trans-3,4-trans-5-d3	0	0

Target Species	Cis-3,5-d2	
Figure	4	
Trial	2	
Isotopic Species	Fraction of Dominant	Percentage Composition
3-d1	1.000	85.6
cis-3,5-d2	0.069	5.9
d0	0.060	5.1
4-d1	0.016	1.4
trans-3,5-d2	0.011	1.0
1-d1	0.007	0.6
cis-3,6-d2	0.002	0.2
1,3-d2	0.002	0.2
cis-3,4-d2	0	0
cis-4,5-d2	0	0
trans-3,4-d2	0	0
trans-4,5-d2	0	0
2,3-d2	0	0
cis-3,4-trans-5-d3	0	0
trans-3,4-trans-6-d3	0	0
trans-3,4-trans-5-d3	0	0

Target Species	Cis-3,5-d2	
Figure	4	
Trial	3	
Isotopic Species	Fraction of Dominant	Percentage Composition
cis-3,5-d2	1.000	78.9
3-d1	0.147	11.6
trans-3,5-d2	0.062	4.9
4-d1	0.020	1.6
trans-3,4-trans-6-d3	0.008	0.6
1,3-d2	0.007	0.5
cis-3,6-d2	0.007	0.5
trans-3,4-d2	0.006	0.5
cis-3,4-d2	0.006	0.5
d0	0.005	0.4
trans-4,5-d2	0	0
cis-4,5-d2	0	0
2,3-d2	0	0
cis-3,4-trans-5-d3	0	0
1-d1	0	0
trans-3,4-trans-5-d3	0	0

Target Species	Cis-3,5-d2	
Figure	4	
Trial	4	
Isotopic Species	Fraction of Dominant	Percentage Composition
cis-3,5-d2	1.000	75.7
3-d1	0.194	14.7
trans-3,5-d2	0.060	4.5
4-d1	0.021	1.6
cis-3,6-d2	0.009	0.7
1,3-d2	0.009	0.7
trans-3,4-trans-6-d3	0.008	0.6
d0	0.008	0.6
trans-3,4-d2	0.007	0.5
cis-3,4-d2	0.006	0.4
trans-4,5-d2	0	0
cis-4,5-d2	0	0
2,3-d2	0	0
cis-3,4-trans-5-d3	0	0
1-d1	0	0
trans-3,4-trans-5-d3	0	0

Target Species	Enantioenriched (S)-3d1	
Figure	6	
Trial	1	
Isotopic Species	Fraction of Dominant	Percentage Composition
3-d1	1.000	89.9
d0	0.053	4.7
1-d1	0.049	4.4
4-d1	0.011	1.0
cis-3,4-d2	0	0
trans-3,4-d2	0	0
cis-3,6-d2	0	0
cis-4,5-d2	0	0
trans-4,5-d2	0	0
cis-3,5-d2	0	0
trans-3,5-d2	0	0
1,3-d2	0	0
2,3-d2	0	0
cis-3,4-trans-5-d3	0	0
trans-3,4-trans-6-d3	0	0
trans-3,4-trans-5-d3	0	0

Target Species	(R)-4d1 Enantioenriched	
Figure	6	
Trial	2	
Isotopic Species	Fraction of Dominant	Percentage Composition
4-d1	1.000	83.1
3-d1	0.070	5.8
d0	0.066	5.5
cis-3,5-d2	0.024	2.0
cis-3,4-d2	0.019	1.6
trans-4,5-d2	0.009	0.7
1-d1	0.006	0.5
trans-3,4-d2	0.004	0.3
2,3-d2	0.004	0.3
cis-3,6-d2	0.003	0.3
trans-3,5-d2	0	0
cis-4,5-d2	0	0
1,3-d2	0	0
cis-3,4-trans-5-d3	0	0
trans-3,4-trans-6-d3	0	0
trans-3,4-trans-5-d3	0	0

Target Species	(R)-4d1 Enantioenriched	
Figure	6	
Trial	3	
Isotopic Species	Fraction of Dominant	Percentage Composition
4-d1	1.000	84.8
3-d1	0.094	8.0
d0	0.059	5.0
1-d1	0.011	0.9
cis-3,4-d2	0.008	0.7
cis-3,6-d2	0.007	0.6
trans-3,4-d2	0	0
cis-4,5-d2	0	0
trans-4,5-d2	0	0
cis-3,5-d2	0	0
trans-3,5-d2	0	0
1,3-d2	0	0
2,3-d2	0	0
cis-3,4-trans-5-d3	0	0
trans-3,4-trans-6-d3	0	0
trans-3,4-trans-5-d3	0	0

Target Species	(R)-3d1 Enantioenriched	
Figure	6	
Trial	4	
Isotopic Species	Fraction of Dominant	Percentage Composition
3-d1	1.000	89.6
d0	0.080	7.2
4-d1	0.017	1.5
1-d1	0.013	1.1
trans-3,4-trans-6-d3	0.006	0.6
trans-3,4-d2	0	0
cis-3,6-d2	0	0
cis-4,5-d2	0	0
trans-4,5-d2	0	0
cis-3,5-d2	0	0
trans-3,5-d2	0	0
1,3-d2	0	0
2,3-d2	0	0
cis-3,4-trans-5-d3	0	0
cis-3,4-d2	0	0
trans-3,4-trans-5-d3	0	0

Target Species	Enantioenriched (R)-4d1	
Figure	6	
Trial	5	
Isotopic Species	Fraction of Dominant	Percentage Composition
4-d1	1.000	70.6
d0	0.319	22.5
3-d1	0.097	6.9
1-d1	0	0
cis-3,4-d2	0	0
trans-3,4-d2	0	0
cis-3,6-d2	0	0
cis-4,5-d2	0	0
trans-4,5-d2	0	0
cis-3,5-d2	0	0
trans-3,5-d2	0	0
1,3-d2	0	0
2,3-d2	0	0
cis-3,4-trans-5-d3	0	0
trans-3,4-trans-6-d3	0	0
trans-3,4-trans-5-d3	0	0

Target Species	Enantioenriched (S)-3d1	
Figure	6	
Trial	6	
Isotopic Species	Fraction of Dominant	Percentage Composition
3-d1	1.000	64.4
4-d1	0.379	24.4
d0	0.109	7.0
1-d1	0.066	4.3
cis-3,4-d2	0	0
trans-3,4-d2	0	0
cis-3,6-d2	0	0
cis-4,5-d2	0	0
trans-4,5-d2	0	0
cis-3,5-d2	0	0
trans-3,5-d2	0	0
1,3-d2	0	0
2,3-d2	0	0
cis-3,4-trans-5-d3	0	0
trans-3,4-trans-6-d3	0	0
trans-3,4-trans-5-d3	0	0

Target Species	(S)-4d1 Enantioenriched
Figure	6
Trial	7
Spectrum File	Enantioenriched 4d1 SPO 10kavg 03_22_23 Vial 288

Isotopic Species	Fraction of Dominant	Percentage Composition
4-d1	1.000	80.6
d0	0.134	10.8
3-d1	0.107	8.7
1-d1	0	0
cis-3,4-d2	0	0
trans-3,4-d2	0	0
cis-3,6-d2	0	0
cis-4,5-d2	0	0
trans-4,5-d2	0	0
cis-3,5-d2	0	0
trans-3,5-d2	0	0
1,3-d2	0	0
2,3-d2	0	0
cis-3,4-trans-5-d3	0	0
trans-3,4-trans-6-d3	0	0
trans-3,4-trans-5-d3	0	0

C) Chiral Analysis of d_1-Cyclohexenes Prepared with Enantioenriched Tungsten Complexes

The chiral tag rotational spectroscopy method[10] to assign the absolute configuration (AC) of the higher abundance enantiomer and measure the enantiomeric excess (EE) of molecules that are chiral by virtue of deuterium substitution has been discussed in previous publications.[11-13] Chiral tag rotational spectroscopy uses chiral derivatization to convert enantiomers, with identical regular rotational spectra, into spectroscopically distinct diastereomers. The chiral tag is small chiral molecule, and it attaches to the analyte through noncovalent interactions during the gas expansion into the spectrometer vacuum system. The chiral tag complexes are analyzed by rotational spectroscopy with identification of the structure guided by quantum chemistry equilibrium geometry calculations. In this work, cyclohexene is complexed with propylene oxide to perform chiral analysis of cyclohexene-3d1 and cyclohexene-4d1.

For enantioisotopomers, the analysis begins with the undeuterated analyte (cyclohexene-d0). Because the equilibrium geometry of all isotopic variants is the same, the geometry of the chiral tag complex can first be determined using the d0-sample and the equilibrium geometry subsequently used to predict the rotational spectrum of any deuterium labeled version of the analyte. The 6-18 GHz rotational spectrum of a 0.1% cyclohexene-d0 and 0.4% propylene oxide gas mixture in neon was acquired for identification of chiral tag complexes with 40K averages. A second, higher sensitivity measurement was performed in the 5-8 GHz frequency region with 970K averages to identify the spectra of the chiral tag complex and its ^{13}C-isotopomers (present in natural abundance). Analysis of the ^{13}C-isotopomers is used to validate the quantum chemistry equilibrium geometry giving exceptionally high confidence for the structure of the chiral tag complex. The rotational spectroscopy fit results are given in Table S4.

Table S4. rotational constants for the cyclopropylene oxide chiral tag complex with the highest spectrum intensity and its ^{13}C isotopomers.

Isotopic Variant[(a)]	Constant	Theory[(b)] (MHz)	Experiment[(c)] (MHz)	% Error[(d)]
Normal Species	A	1873.42	1876.6846(77)	0.1743
	B	772.94	746.65580(22)	-3.4005
	C	710.69	689.02411(26)	-3.0486
13C-1	A	1860.65	1860.6622(15)	0.0000
	B	743.81	743.92426(75)	0.0154
	C	685.78	685.78686(58)	0.0010
13C-2	A	1863.43	1863.4847(35)	0.0029
	B	741.84	741.81094(68)	-0.0039
	C	683.57	683.63124(62)	0.0090
				--
13C-3	A	1863.62	1863.6622(14)	0.0023
	B	741.89	742.05985(62)	0.0229
	C	688.76	686.85806(66)	-0.2761
13C-4	A	1863.14	1863.1399(11)	0.0000
	B	742.05	742.12516(49)	0.0101
	C	684.22	684.24185(52)	0.0032
13C-5	A	1862.68	1862.7137(24)	0.0018
	B	743.17	743.13445(73)	-0.0048
	C	685.15	685.19540(60)	0.0066
13C-6	A	1862.10	1862.1138(18)	0.0001
	B	742.27	742.33112(56)	0.0082
	C	687.30	687.30890(49)	0.0013
13C-17	A	1873.10	1873.1957(14)	0.0051
	B	735.43	735.49647(48)	0.0090
	C	679.00	679.06175(46)	0.0091
13C-19	A	1875.22	1875.2299(16)	0.0001
	B	741.39	741.43904(60)	0.0066
	C	684.66	684.69008(55)	0.0044
13C-22	A	1859.02	1859.1591(15)	0.0075
	B	741.88	741.97207(64)	0.0124
	C	682.59	682.73024(57)	0.0205

(a) The labeling of the carbon atom uses the atom numbering from the quantum chemistry geometry optimization. This calculation can be found in the Zenodo data archive.
(b) The theory results reported for the normal species are the rotational constants calculated from the quantum chemistry equilibrium geometry. For the ^{13}C-isotopomers, the theory values are the scaled predictions using this equilibrium geometry where the calculated values are corrected using the normal species (NS) results. For example, the scale factor for the prediction of the A rotational constant of all ^{13}C-isotpoomers is: $A_{scale} = (A^{NS}_{exp} / A^{NS}_{theory})$.
(c) The values in parenthesis are the 1σ fit uncertainties in the last two digits.
(d) The % Error is calculated using the experimental minus the theoretical values.

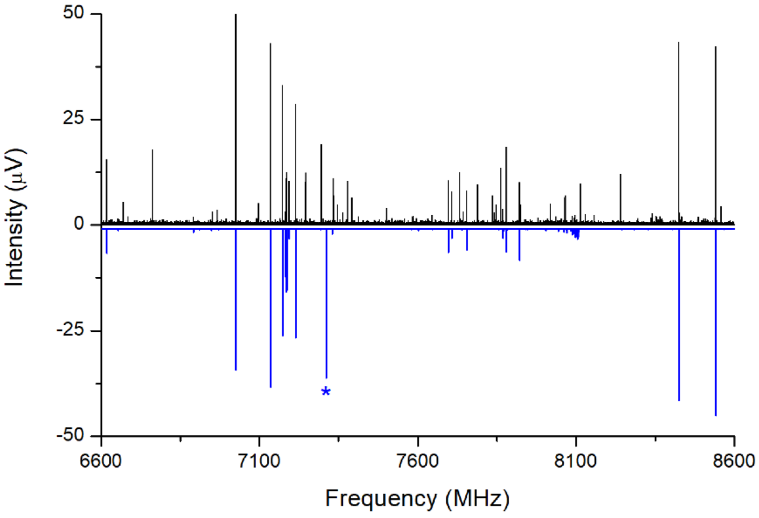

Figure S6: A section of the 6-18 GHz MRR spectrum of a gas mixture of 0.1% cyclohexene and 0.4% propylene oxide in neon is shown in black. The spectrum of the complex formed between cyclohexene and propylene oxide that is used for chiral analysis is shown in blue. This spectrum is calculated from the fit rotational constants in Table S4. For clarity, the experimental spectrum has the known transitions associated with cyclohexene and propylene oxide (uncomplexed) cut. The transition marked with the asterisk is missing from the experimental spectrum because it overlaps with a transition of propylene oxide.

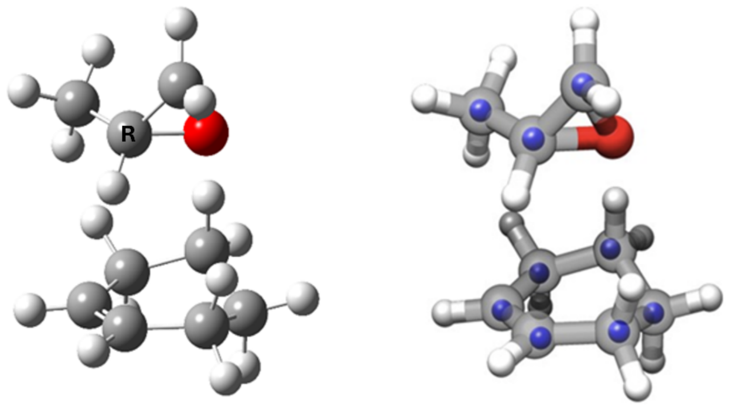

Figure S7: The equilibrium geometry of the chiral tag complex between cyclohexene and (*R*)-propylene oxide associated with the spectrum in Figure S6 is shown on the left. This structure was calculate using the B3LYP GD3BJ def2TZVP model chemistry. The experimental carbon atom positions of Table S5 are shown superimposed on the equilibrium geometry in the structure on the right. The signs for the carbon atom coordinates are chosen by comparison between the Kraitchman coordinates and the quantum chemistry structure.

A small frequency range of the 6-18 GHZ rotational spectrum of the cyclohexene / propylene oxide gas mixture is shown in Figure S6. The figure also shows the calculated MRR spectrum of the highest intensity complex formed between cyclohexene and propylene oxide. This calculated spectrum uses the fit rotational constants given in Table S3 and the dipole moment vector components from the quantum chemistry equilibrium geometry. The equilibrium geometry is calculated in Gaussian 16[14] using dispersion corrected density functional theory[15] and the model chemistry: B3LYP GD3BJ def2TZVP. This geometry is shown in Figure S7. The 9 ^{13}C-isotopomer spectra are also assigned, and their rotational spectroscopy fit constants are also reported in Table S4. These fit constants are used to calculate postion information for each carbon atom in the principal axis system of molecular rotation using the method of Kraitchman.[16] This method converts the changes in the moments-of-inertia upon isotopic substitution at a single atom position into the magnitude of the atom coordinate positions of the "normal species" structure (the structure formed from the nuclei with highest natural abundance for each element). The sign of the coordinate position cannot be determined because the moments-of-inertia depend on the coordinate value squared. The experimental magnitude carbon atom coordinates are compared to the carbon atom positions in the

equilibrium geometry in Table S5. The excellent agreement in the atom positions gives exceptionally high-confidence that the equilibrium geometry of Figure S7 gives rise to the experimental rotational spectrum in Figure S6.

Table S5. Kraichman carbon atom parameters for the cyclohexene-propylene oxide chiral tag complex.

Carbon Atom[(a)]	Coordinate[(b)]	Theory (Å)	Experiment[(c)] (Å)
13C-1	a	1.330	1.3530 ± 0.0011
	b	1.260	1.2882 ± 0.0012
	c	0.870	0.8386 ± 0.0018
13C-2	a	1.999	2.0471 ± 0.0008
	b	1.300	1.2919 ± 0.0012
	c	-0.506	0.534 ± 0.003
13C-3	a	1.526	1.5283 ± 0.0010
	b	0.135	0.04 ± 0.04
	c	-1.374	1.3847 ± 0.0011
13C-4	a	1.882	1.9200 ± 0.0008
	b	-1.206	1.2239 ± 0.0012
	c	-0.728	0.711 ± 0.002
13C-5	a	1.588	1.6336 ± 0.0009
	b	-1.222	1.2130 ± 0.0013
	c	0.746	0.768 ± 0.002
13C-6	a	1.329	1.3607 ± 0.0011
	b	-0.125	0.00**
	c	1.454	1.4660 ± 0.0010
13C-17	a	-3.163	3.225 ± 0.005
	b	0.729	0.718 ± 0.002
	c	0.029	0.07 ± 0.02
13C-19	a	-2.121	2.1594 ± 0.0011
	b	-0.227	0.2138 ± 0.012
	c	0.403	0.410 ± 0.006
13C-22	a	-2.040	2.0695 ± 0.0007
	b	-1.602	1.6049 ± 0.0010
	c	-0.190	0.163 ± 0.009

(a) The labeling of the carbon atom uses the atom numbering from the quantum chemistry geometry optimization. This calculation can be found in the Zenodo data archive.
(b) The atom coordinate in the principal axis system of the normal species.
(c) The errors include the Costain error estimate.
** This coordinate value can't be determined due to a large inertial defect. This effect is commonly observed in the Kraitchman method and generally indicates that the coordinate value is small. The value is reported as zero to indicate that a small value is expected.

Using the quantum chemistry equilibrium geometry of Figure S7, the rotational spectra of any deuterated cyclohexene complexed to propylene oxide can be calculated to high accuracy leading to rapid analysis of the chiral tag spectrum of the deuterated analyte. In this work, chiral tag rotational spectroscopy is used to analyze cyclohexene-3d1 and cyclohexene-4d1. When a racemic sample cyclohexene-d1 and enantiopure (S)-propylene oxide ((S)-PO) is measured, four chiral tag rotational spectra are expected. The resulting chiral tag complexes are either homochiral or heterochiral where a homochiral complex is formed from complexation of (S)-cyclohexene-3d1 and (S)-PO in this example. As discussed in section B, the transient chirality of the ring pucker gives two diastereomers of (S)-cyclohexene-3d1 so that two homochiral tag complexes are formed. The four chiral tag complexes formed between cyclohexene-3d1 and (S)-PO are shown in Figure S8. Since these structures have a single isotopic substitution (H-to-D), the atom position for the substitution can be calculated by Kraitchman's method to verify the deuterium position associated with each of the four experimental spectra. A summary of the rotational spectroscopy analysis of the four chiral tag isomers for cyclohexene-3d1 and four isomers for cyclohexene-4d1 is presented in Table S6. The agreement of the experimental Kraitchman coordinates with the hydrogen atom positions in the equilibrium geometry gives high confidence for the assignment of the four spectra to respective homochiral or heterochiral complexes.

An example of using chiral tag rotational spectroscopy to establish the absolute configuration of the higher abundance enantiomer of an enantioenriched sample of cyclohexene-3d1. This sample is reported as Trial 1 in Figure 6 of the main paper. The top panel of Figure S9 shows the measured spectrum of a racemic sample of cyclohexene-3d1 with (S)-PO tag sample in a 600 MHz region of the full 2-8 GHz spectrum acquisition. The highest intensity transitions are from the uncomplexed cyclohexene-3d1. These transitions are assigned to the ring pucker isomers, see Table S2. The rotational spectra of the four chiral tag complexes of Figure S8 are identified by showing the color-coded spectrum simulations calculated using the fit rotational constants of Table S6. Note that all four spectra have equal intensity. For a racemic sample

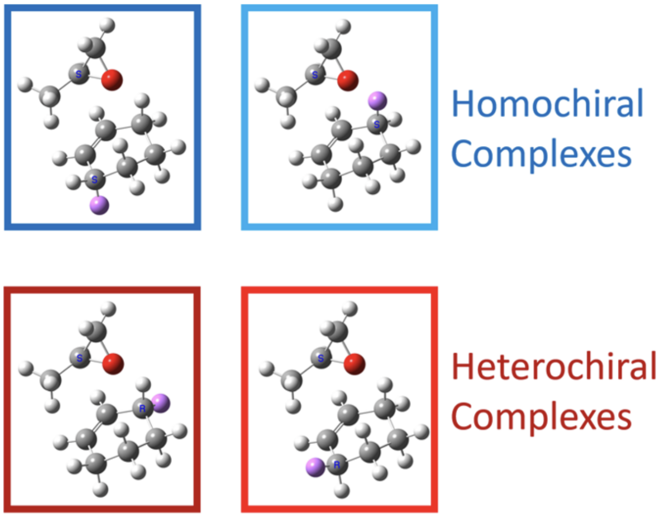

Figure S8: The four isomers of the chiral tag complex formed between cyclohexene-3d1, and (S)-PO are shown. The homochiral complexes are formed by the complexation of (S)-cyclohexene-3d1 and (S)-PO. The heterochiral complex form between (R)-cyclohexene-3d1 and (S)-PO. The two isomers in each family come from the propylene oxide attaching to opposite faces of the cyclohexene-3d1 ring.

where the cyclohexene-3d1 enantiomers are present in equal amounts, the total intensity of homochiral and heterochiral spectrum intensity is expected to be equal. In addition, the ring pucker isomers of each enantiomer are expected to be in equal abundance so that the two homochiral (and two heterochiral) chiral tag complex spectra are expected to have equal intensity.

The next two spectra of Figure S9 show rotational transitions in a small frequency range of the 2-8 GHz measurement for the homochiral and heterochiral tag complexes of cyclohexene-3d1. The measurement using a racemic sample of cyclohexene-3d1 is shown in the middle panel. A single transition from the rotational spectra of the four chiral tag isomers in Figure S8 falls in this window. All four transitions have approximately equal intensity in the racemic measurement. This measurement is used to calibrate the instrument response. The bottom panel shows the MRR measurement using the enantioenriched cyclohexene-3d1 sample and enantiopure (S)-PO tag sample. In this case, the transitions in the rotational spectra of the two homochiral tag complexes are higher relative intensity when (S)-PO is used as the tag. This indicates that (S)-cyclohexene-3d1 is the absolute configuration of the higher abundance enantiomer from the synthesis.

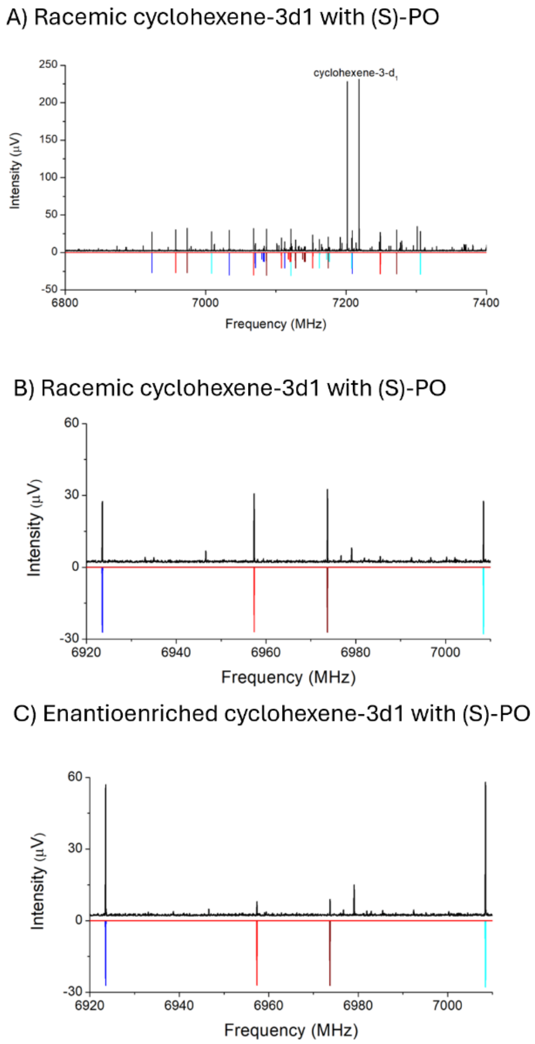

Figure S9: A region of the 2-8 GHz MRR spectrum of a gas mixture of racemic cyclohexene-3d1 and (S)-PO is shown in A and B. Spectra assigned to all four complexes in Figure S8 are observed. The spectra calculated from the fit rotational constants are color-coded using the scheme in Figure S8. The spectrum acquired using enantioenriched cyclohexene-3d1 is shown in C.

Table S6 : The Fit Rotational Constants and Kraitchman Coordinates for the Four d1-Isotpomers of the Cyclohexene-3d1 – Propylene Oxide and Cyclohexene-4d1 – Propylene Oxide Chiral Tag Complexes

a) Cyclohexene-3d1 / Propylene Oxide Chiral Tag Complex

Isotopomer(a)	Constant	Theory(b) (MHz)	Experiment (MHz)	% Error	Coordinate	Theory (Å)	Expt (Å)
D10 HOM1	A	1854.40	1855.532(51)	0.061	a	0.298	0.00**
	B	745.88	746.65067(29)	0.103	b	1.620	1.629
	C	686.38	686.89590(29)	0.075	c	0.787	0.649
D12 HOM2	A	1857.57	1857.733(71)	0.009	a	2.944	2.971
	B	736.01	736.21131(37)	0.027	b	-1.433	1.427
	C	678.76	678.93818(37)	0.026	c	-0.886	0.875
D11 HET1	A	1834.84	1834.693(54)	-0.008	a	1.836	1.839
	B	740.24	740.59868(31)	0.048	b	1.945	1.985
	C	682.01	682.06455(31)	0.008	c	1.558	1.510
D13 HET2	A	1838.95	1838.802(45)	-0.008	a	1.337	1.322
	B	743.03	743.31232(27)	0.038	b	-2.019	2.049
	C	683.30	683.39529(27)	0.014	c	-1.220	1.173

a) Cyclohexene-4d1 / Propylene Oxide Chiral Tag Complex

Isotopomer(a)	Constant	Theory(b) (MHz)	Experiment (MHz)	% Error	Coordinate	Theory (Å)	Expt (Å)
D7 HOM	A	1838.42	1837.898(80)	-0.028	a	1.965	1.921
	B	735.99	736.43256(35)	0.060	b	0.197	0.00**
	C	685.22	685.53945(35)	0.047	c	-2.373	2.391
				--			
D9 HOM	A	1835.77	1835.811(58)	0.002	a	1.788	1.854
	B	741.95	741.75277(35)	-0.027	b	2.252	2.230
	C	681.03	681.13619(35)	0.016	c	-0.997	1.043
				--			
D8 HET	A	1865.46	1865.85(11)	0.021	a	3.086	3.118
	B	735.75	735.87359(58)	0.017	b	1.239	1.210
	C	678.48	678.69621(58)	0.032	c	-0.381	0.418
				--			
D16 HET	A	1860.98	1861.04(14)	0.003	a	0.442	0.00**
	B	743.89	745.42605(72)	0.206	b	0.205	0.267
	C	688.79	689.98918(72)	0.174	c	-1.496	1.489

(a) The labeling of the hydrogen/deuterium atom uses the atom numbering from the quantum chemistry geometry optimization. This calculation can be found in the Zenodo data archive.
(b) The theory values are the scaled predictions using this equilibrium geometry where the calculated values are corrected using the normal species (NS) results in Table MRR-3. For example, the scale factor for the prediction of the A rotational constant of all D-isotopomers is: $A_{scale} = (A^{NS}_{exp} / A^{NS}_{theory})$.
** This coordinate value can't be determined due to a large inertial defect. This effect is commonly observed in the Kraitchman method and generally indicates that the coordinate value is small. The value is reported as zero to indicate that a small value is expected.

The relative intensities of the homochiral and heterochiral transitions can be used to measure the enantiomeric excess in the chiral tag rotational spectroscopy analysis.[11-12] The racemic measurements are used to correct the instrument response and normalize the measured transition intensity. For each transition observed in the chiral tag rotational spectrum, a normalized signal intensity is calculated:

$$N_{HOM} = \frac{I_{HOM}^{enriched}}{I_{HOM}^{racemic}} \quad (1)$$

$$N_{HET} = \frac{I_{HET}^{enriched}}{I_{HET}^{racemic}} \quad (2)$$

For any pair of homochiral and heterochiral transitions, the normalized signals are used to calculate the ratio, R, which is approximately the enantiomer ratio:

$$R = \frac{N_{HOM}}{N_{HET}} \quad (3)$$

This ratio is related to the enantiomeric excess of the analyte by:

$$\frac{R-1}{R+1} = \left(ee_{cyclohexene-d1}\right)\left(ee_{tag}\right) \quad (4)$$

In Eq. (10), *ee* denotes the enantiomeric excess as a fractional value instead of percent. The enantiomeric excess determination is corrected for the measured enantiopurity of the tag sample in separate chiral tag rotational spectroscopy measurement: ee_{tag} = 0.996 for (S)-PO.

The enantiomeric excess is reported as the percent EE of the analyte:

$$\mathrm{EE} = \left(ee_{cyclohexene-d1}\right) \times 100 \quad (5)$$

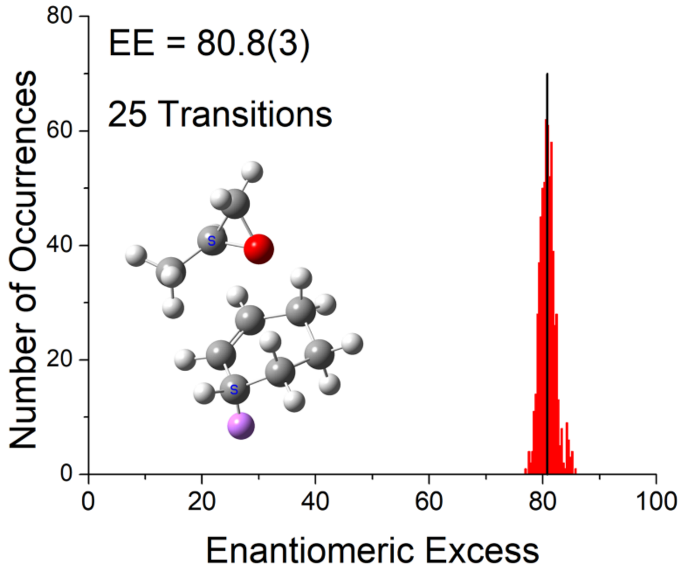

Figure S10: The histogram of 625 pairwise EE detemrinations using the 25 highest intensity transitions in the MRR spectra of the homochiral and heterochiral tag complexes of Figure S8 is shown.

The broadband spectrum is used to determine the enantiomeric and estimate the measurement precision. For the spectrum measurement in Figure S9, this process uses the 25 highest intensity transitions from both the homochiral and heterochiral spectra (using both isomers as shown in Figure S8). (The number of transitions used in the calculation depends on the signal-to-noise ratio of the chiral tag MRR measurement.) These transitions are used to calculate 625 EE values using Eqs. (1)-(4). A histogram of the 625 EE determinations of the cyclohexene-3d1 sample is shown in Figure S10. Note that the EE values are positive and this comes from the definition of the signal ratio, R, in Eq. (3). If the sample had been the other enantiomer ((R)-cyclohexene-3d1), then R<1 and the EE values would be negative. In this way, the sign of the EE values reported below indicate the absolute configuration of the higher abundance enantiomer. The sample EE is the mean value of all 625 determinations, and the measurement precision is estimated by the standard error. For the enantioenriched (S)-cyclohexene-3d1 sample, the enantiomeric excess is EE = 80.8(3) by chiral tag rotational spectroscopy (the value in parenthesis is the 1σ estimate of the measurement reproducibility).

The histogram results (Figure S11) for the seven samples analyzed in this work, reported in Figure 6 of the main paper, are shown below:

Figure S11. Histogram results for the seven samples analyzed.

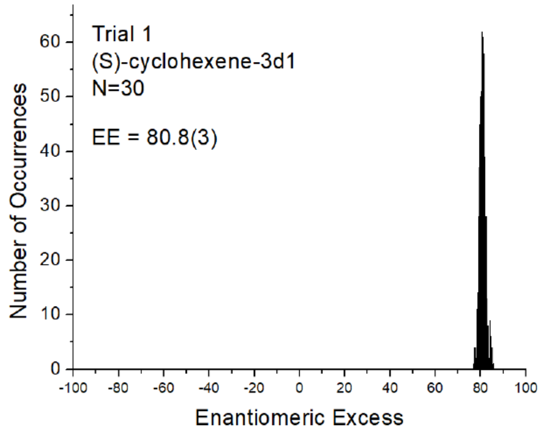

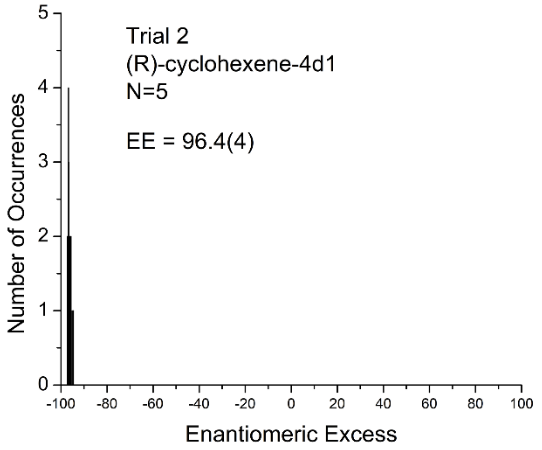
Trial 2
(R)-cyclohexene-4d1
N=5
EE = 96.4(4)
Number of Occurrences
Enantiomeric Excess

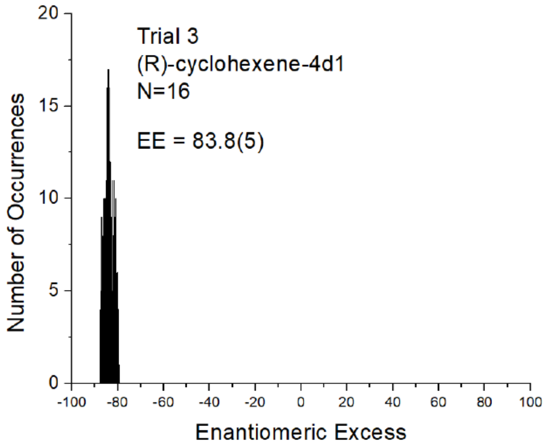
Trial 3
(R)-cyclohexene-4d1
N=16
EE = 83.8(5)
Number of Occurrences
Enantiomeric Excess

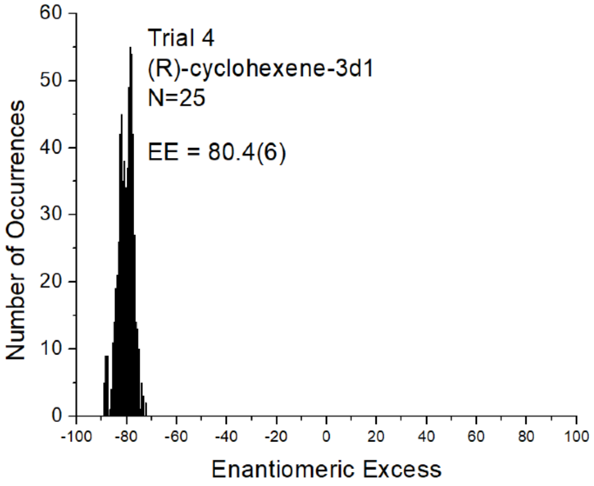
Trial 4
(R)-cyclohexene-3d1
N=25
EE = 80.4(6)
Number of Occurrences
Enantiomeric Excess

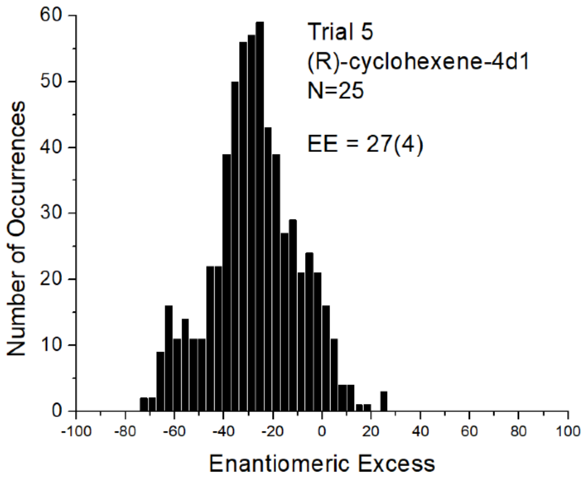
Trial 5
(R)-cyclohexene-4d1
N=25
EE = 27(4)
Number of Occurrences
Enantiomeric Excess

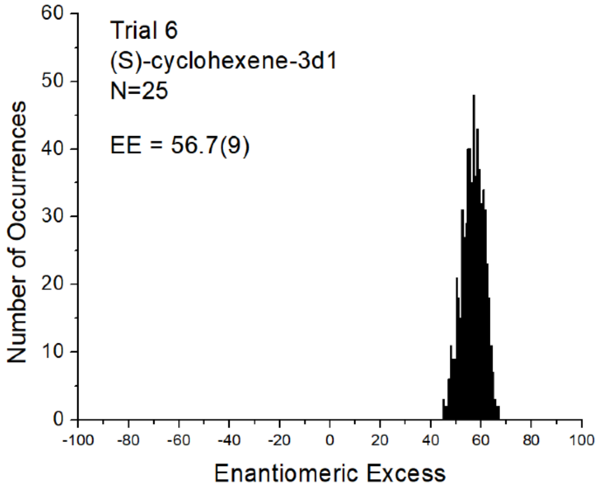
Trial 6
(S)-cyclohexene-3d1
N=25
EE = 56.7(9)
Number of Occurrences
Enantiomeric Excess

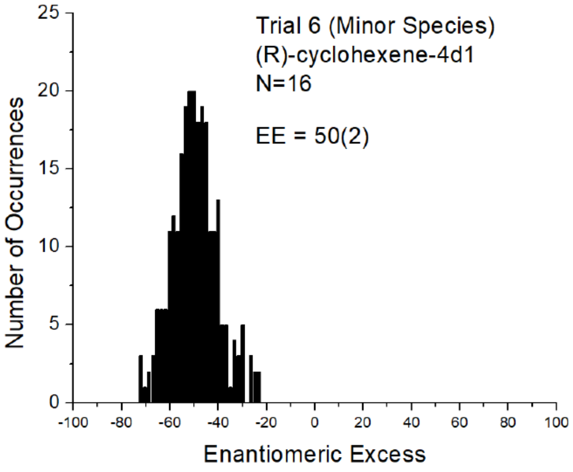
Trial 6 (Minor Species)
(R)-cyclohexene-4d1
N=16
EE = 50(2)
Number of Occurrences
Enantiomeric Excess

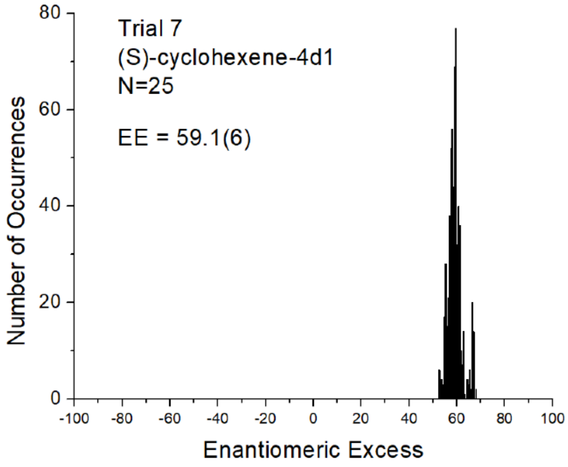
Trial 7
(S)-cyclohexene-4d1
N=25
EE = 59.1(6)
Number of Occurrences
Enantiomeric Excess

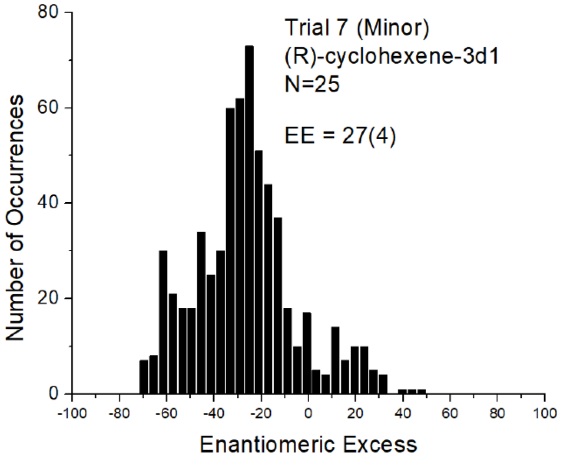
Trial 7 (Minor)
(R)-cyclohexene-3d1
N=25
EE = 27(4)
Number of Occurrences
Enantiomeric Excess

Crystallographic Data

A single crystal of (*S*)-4-*exo*-d_1-**4D** was coated with Paratone oil and mounted on a MiTeGen MicroLoop. The X-ray intensity data were measured on a Bruker Kappa APEXII Duo system equipped with a fine-focus sealed tube (Mo K_α, λ = 0.71073 Å) and a graphite monochromator. The frames were integrated with the Bruker SAINT software package[17] using a narrow-frame algorithm. Data were corrected for absorption effects using the Multi-Scan method (SADABS).[18] The structure was solved and refined using the Bruker SHELXTL Software Package[19] within APEX3[17] and OLEX2.[20] Non-hydrogen atoms were refined anisotropically. The B-H hydrogen atoms, as well as the allylic hydrogens H10-H12 and H29-H31 were located in the electron density map and refined isotropically. All other hydrogen atoms were placed in geometrically calculated positions with $U_{iso} = 1.2U_{equiv}$ of the parent atom ($1.5U_{equiv}$ for methyl). Anomalous dispersion was used to determine the absolute structure parameter and confirm the absolute stereochemistry of the molecule.

	(*S*)-4-*exo*-d_1-**4D**
CCDC number	2350579
Formula	$C_{19}H_{28}BF_3N_7O_4PSW$
FW (g/mol)	733.17
Temp (K)	100(2)
λ (Å)	0.71073
Size (mm)	0.156 x 0.320 x 0.418
Crystal habit	yellow-orange block
Crystal system	monoclinic
Space group	C 2
a (Å)	26.725(3)
b(Å)	11.5860(11)
c (Å)	17.0394(17)
α (°)	90
β (°)	95.411(2)
γ (°)	90
Volume (Å^3)	5252.5(9)
Z	8
Density (g/cm^3)	1.854
μ (mm^{-1})	4.601
F(000)	2880
θ range (°)	1.20 to 33.19
Index ranges	$-41 \leq h \leq 41$<br>$-17 \leq k \leq 17$<br>$-24 \leq l \leq 26$
Reflns collected	108103
Independent reflns	20065 [R_{int} = 0.0340]
Data / restraints /parameters	20065 / 1 / 705
GOF on F^2	1.032
R_1 (I>2σ(I))	0.0211
wR_2 (all data)	0.0460

Absolute structure parameter	-0.015(3)

Table S7. Crystallographic parameters for (*S*)-4-*exo*-d_1-**4D**.

Computational Methods:
Structures were located with Gaussian 16[21] with the M06 functional and 6-31G** basis set for all atoms except W, where the LANL2DZ basis set/pseudopotential was used. Previous literature demonstrates that this functional/basis set pair corroborates experimental results.[22] Solvent effects of acetonitrile were modeled using SMD. Gaussian's default criteria were used for optimization, vibrational frequency analysis verified that minima had all positive frequencies and transition states have a single negative frequency. Gibbs free energy values correspond to SCF electronic energies with addition of the typical rigid-rotor harmonic oscillator thermochemical correction at 298 K and 1 atm.

Compound	Electronic Energy (Hartree)	Relative Free Energy (kcal/mol)
$6H^D$	-1593.992964	0.0 (ref)
TS ($6H^D \rightarrow$ 4D)	-1593.964422	+17.9
4D	-1594.016150	-14.6
$6H^P$	-1593.994349	0.0 (ref)
TS ($6H^P \rightarrow$ 4P)	-1593.965188	+18.3
4P	-1594.014040	-12.4
$6H^D$	-1593.992964	+14.6
4D	-1594.016150	0.0 (ref)
$6H^P$	-1593.994349	+13.7
4P	-1594.014040	+1.3

Table S8. Summary of ground and transition state free energies calculated for **6H**, **4D**, and **4P**.

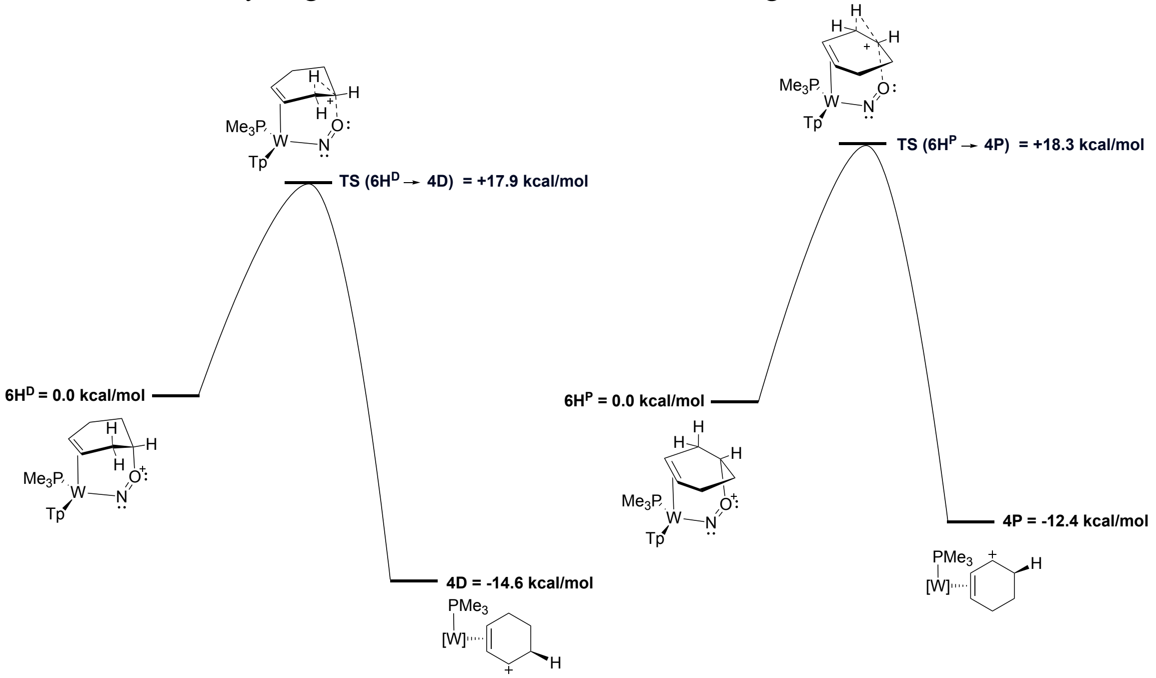

Figure S12. Reaction coordinate diagram supporting Figure 4 in manuscript.

References

1. Smith, J. A.; Wilson, K. B.; Sonstrom, R. E.; Kelleher, P. J.; Welch, K. D.; Pert, E. K.; Westendorff, K. S.; Dickie, D. A.; Wang, X.; Pate, B. H.; Harman, W. D., Preparation of cyclohexene isotopologues and stereoisotopomers from benzene. *Nature* **2020,** *581* (7808), 288-293.
2. Lankenau, A. W.; Iovan, D. A.; Pienkos, J. A.; Salomon, R. J.; Wang, S.; Harrison, D. P.; Myers, W. H.; Harman, W. D., Enantioenrichment of a Tungsten Dearomatization Agent Utilizing Chiral Acids. *J. Am. Chem. Soc.* **2015,** *137* (10), 3649-3655.
3. Wilson, K. B.; Smith, J. A.; Nedzbala, H. S.; Pert, E. K.; Dakermanji, S. J.; Dickie, D. A.; Harman, W. D., Highly Functionalized Cyclohexenes Derived from Benzene: Sequential Tandem Addition Reactions Promoted by Tungsten. *The Journal of Organic Chemistry* **2019,** *84* (10), 6094-6116.
4. Harrison, D. P.; Nichols-Nielander, A. C.; Zottig, V. E.; Strausberg, L.; Salomon, R. J.; Trindle, C. O.; Sabat, M.; Gunnoe, T. B.; Iovan, D. A.; Myers, W. H.; Harman, W. D., Hyperdistorted Tungsten Allyl Complexes and Their Stereoselective Deprotonation to Form Dihapto-Coordinated Dienes. *Organometallics* **2011,** *30* (9), 2587-2597.
5. Brown, G. G.; Dian, B. C.; Douglass, K. O.; Geyer, S. M.; Shipman, S. T.; Pate, B. H., A broadband Fourier transform microwave spectrometer based on chirped pulse excitation. *Rev Sci Instrum* **2008,** *79* (5), 053103.
6. Pérez, C.; Lobsiger, S.; Seifert, N. A.; Zaleski, D. P.; Temelso, B.; Shields, G. C.; Kisiel, Z.; Pate, B. H., Broadband Fourier transform rotational spectroscopy for structure determination: The water heptamer. *Chemical Physics Letters* **2013,** *571*, 1-15.
7. Vang, Z. P.; Reyes, A.; Sonstrom, R. E.; Holdren, M. S.; Sloane, S. E.; Alansari, I. Y.; Neill, J. L.; Pate, B. H.; Clark, J. R., Copper-Catalyzed Transfer Hydrodeuteration of Aryl Alkenes with Quantitative Isotopomer Purity Analysis by Molecular Rotational Resonance Spectroscopy. *J. Am. Chem. Soc.* **2021,** *143* (20), 7707-7718.
8. Gordy, W.; Cook, R. L., *Microwave Molecular Spectra*. 3rd ed.; John Wiley & Sons, Inc.: New York, 1984.
9. Snow, M. S.; Howard, B. J.; Evangelisti, L.; Caminati, W., From Transient to Induced Permanent Chirality in 2-Propanol upon Dimerization: A Rotational Study. *The Journal of Physical Chemistry A* **2011,** *115* (1), 47-51.
10. Pate, B. H.; Evangelisti, L.; Caminati, W.; Xu, Y.; Thomas, J.; Patterson, D.; Perez, C.; Schnell, M., Chapter 17 - Quantitative Chiral Analysis by Molecular Rotational Spectroscopy. In *Chiral Analysis (Second Edition)*, Polavarapu, P. L., Ed. Elsevier: 2018; pp 679-729.
11. Mills, M. D.; Sonstrom, R. E.; Vang, Z. P.; Neill, J. L.; Scolati, H. N.; West, C. T.; Pate, B. H.; Clark, J. R., Enantioselective Synthesis of Enantioisotopomers with Quantitative Chiral Analysis by Chiral Tag Rotational Spectroscopy. *Angewandte Chemie International Edition* **2022,** *61* (33), e202207275.
12. Sonstrom, R. E.; Vang, Z. P.; Scolati, H. N.; Neill, J. L.; Pate, B. H.; Clark, J. R., Rapid Enantiomeric Excess Measurements of Enantioisotopomers by Molecular Rotational Resonance Spectroscopy. *Organic Process Research & Development* **2023**.
13. Vang, Z. P.; Sonstrom, R. E.; Scolati, H. N.; Clark, J. R.; Pate, B. H., Assignment of the absolute configuration of molecules that are chiral by virtue of deuterium substitution using chiral tag molecular rotational resonance spectroscopy. *Chirality* **2023,** *35* (11), 856-883.
14. M. J. Frisch, G. W. T., H. B. Schlegel, G. E. Scuseria, M. A. Robb, J. R. Cheeseman, G. Scalmani, V. Barone, G. A. Petersson, H. Nakatsuji, X. Li, M. Caricato, A. V. Marenich, J. Bloino, B. G. Janesko, R. Gomperts, B. Mennucci, H. P. Hratchian, J. V. Ortiz, A. F. Izmaylov, J. L. Sonnenberg, D. Williams-Young, F. Ding, F. Lipparini, F. Egidi, J. Goings, B. Peng, A. Petrone, T. Henderson, D. Ranasinghe, V. G. Zakrzewski, J. Gao, N. Rega, G. Zheng, W. Liang, M. Hada, M. Ehara, K. Toyota, R. Fukuda, J. Hasegawa, M. Ishida, T. Nakajima, Y. Honda, O. Kitao, H. Nakai, T. Vreven, K. Throssell, J. A. Montgomery, Jr., J. E. Peralta, F. Ogliaro, M. J. Bearpark, J. J. Heyd, E. N. Brothers, K. N. Kudin, V. N. Staroverov, T. A. Keith, R. Kobayashi, J. Normand, K. Raghavachari,

A. P. Rendell, J. C. Burant, S. S. Iyengar, J. Tomasi, M. Cossi, J. M. Millam, M. Klene, C. Adamo, R. Cammi, J. W. Ochterski, R. L. Martin, K. Morokuma, O. Farkas, J. B. Foresman, and D. J. Fox *Gaussian 16, Revision B.01*, Gaussian, Inc.: Wallingford CT, 2016.

15. Grimme, S.; Ehrlich, S.; Goerigk, L., Effect of the damping function in dispersion corrected density functional theory. *J Comput Chem* **2011,** *32* (7), 1456-65.
16. Kraitchman, J., Determination of Molecular Structure from Microwave Spectroscopic Data. *American Journal of Physics* **1953,** *21* (1), 17-24.
17. Bruker (2019). Saint; APEX3. Bruker AXS Inc., Madison, Wisconsin, USA.
18. Krause, L.; Herbst-Irmer, R.; Sheldrick, G. M.; Stalke, D. Comparison of silver and molybdenum microfocus X-ray sources for single-crystal structure determination *J. Appl. Cryst* **2015**, 48, 3-10. doi:10.1107/ S1600576714022985
19. Sheldrick, G. M. SHELXT – Integrated space-group and crystal-structure determination *Acta Cryst* **2015**, A71, 3-8.
20. Dolomanov, O. V.; Bourhis, L. J.; Gildea, R. J.; Howard, J. A. K.; Puschmann, H. *OLEX2*: a complete structure solution, refinement and analysis program *J. Appl. Cryst* **2009**, 42, 339-341.
21. Frisch, M. J.; Trucks, G. W.; Schlegel, H. B.; Scuseria, G. E.; Robb, M. A.; Cheeseman, J. R.; Scalmani, G.; Barone, V.; Petersson, G. A.; Nakatsuji, H.; Li, X.; Caricato, M.; Marenich, A. V.; Bloino, J.; Janesko, B. G.; Gomperts, R.; Mennucci, B.; Hratchian, H. P.; Ortiz, J. V.; Izmaylov, A. F.; Sonnenberg, J. L.; Williams; Ding, F.; Lipparini, F.; Egidi, F.; Goings, J.; Peng, B.; Petrone, A.; Henderson, T.; Ranasinghe, D.; Zakrzewski, V. G.; Gao, J.; Rega, N.; Zheng, G.; Liang, W.; Hada, M.; Ehara, M.; Toyota, K.; Fukuda, R.; Hasegawa, J.; Ishida, M.; Nakajima, T.; Honda, Y.; Kitao, O.; Nakai, H.; Vreven, T.; Throssell, K.; Montgomery Jr., J. A.; Peralta, J. E.; Ogliaro, F.; Bearpark, M. J.; Heyd, J. J.; Brothers, E. N.; Kudin, K. N.; Staroverov, V. N.; Keith, T. A.; Kobayashi, R.; Normand, J.; Raghavachari, K.; Rendell, A. P.; Burant, J. C.; Iyengar, S. S.; Tomasi, J.; Cossi, M.; Millam, J. M.; Klene, M.; Adamo, C.; Cammi, R.; Ochterski, J. W.; Martin, R. L.; Morokuma, K.; Farkas, O.; Foresman, J. B.; Fox, D. J. Gaussian 16 Rev. C.01, Wallingford, CT, 2016.
22. Smith, J.A., Schouten, A., Wilde, J.H., Westendorff, K.S., Dickie, D.A., Ess, D.H., Harman, W.D. Experiments and Direct Dynamics Simulations That Probe η^2-Arene/Aryl Hydride Equilibria of Tungsten Benzene Complexes. *J Am Chem Soc* **2020**, 142, 38, 16437–16454.
